# Experimental Evidence for a Metal‐Related Function of a Cyanobactin

**DOI:** 10.1002/anie.8567749

**Published:** 2026-05-02

**Authors:** Philipp Baur, Timothy A. Hill, Kai‐Chen Wu, Junxian Lim, Brett M. Paterson, Gunasekaran Velmurugan, Bernard M. Degnan, David P. Fairlie, Peter Comba

**Affiliations:** ^1^ Universität Heidelberg Anorganisch‐Chemisches Institut, and Interdisciplinary Center for Scientific Computing (IWR) Heidelberg Germany; ^2^ Max Planck School Matter to Life Heidelberg Germany; ^3^ Institute for Molecular Bioscience The University of Queensland Brisbane Queensland Australia; ^4^ ARC Centre of Excellence for Innovations in Peptide and Protein Science Institute for Molecular Bioscience The University of Queensland Brisbane Queensland Australia; ^5^ Centre for Advanced Imaging Australian Institute for Bioengineering and Nanotechnology The University of Queensland, St. Lucia Queensland Australia; ^6^ Department of Chemistry National Institute of Technology – Tiruchirappalli Tiruchirappalli Tamil Nadu India; ^7^ Centre for Marine Science and School of the Environment The University of Queensland, St. Lucia Queensland Australia

## Abstract

The cyanobacterium *Prochloron didemni* produces macrocyclic octapeptides with thiazole and oxazoline heterocycles, known as patellamides. An interesting observation is that Cu^2+^ binding to the patellamides is likely to be related to their biological function. First, we show that Cu^2^^+^ injection into *Lissoclinum patella* increases *patG* gene expression and patellamide levels in the ascidians. Second, x‐ray absorption spectroscopy shows that biological extracts of specimen from the Great Barrier Reef match structurally synthetic carbonato‐bridged dicopper(II)‐patellamide complexes. Third, patellamides exhibit very high membrane permeability (PAMPA, Caco‐2). Combined with intracellular pH data, patellamide‐Cu^2^^+^ bioactivity in algae, and the absence of many of the typical CO_2_ uptake mechanisms in *Prochloron*, we propose that patellamides facilitate carbonate transport from the ascidians to the cyanobacteria. This provides unprecedented evidence for a link between cyanobactin metal binding and their production and function, suggesting possible novel metal‐related roles for marine cyclic peptides.

## Graphical abstract

Cyanobactins such as patellamides are produced by cyanobacteria profusely and have been studied extensively, but almost exclusively for their cytotoxic properties. Discussed is unprecedented experimental evidence for a metal‐related biological function – likely CO_2_ transport from the ascidian to the cyanobacterial symbiont. Beyond cytotoxicity, nature may have embedded sophisticated metal chemistry into many more natural products yet to be explored.

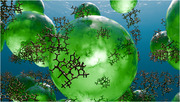

## 1 Introduction

The patellamides are a class of cyclic pseudo‐octapeptides with a relatively rigid backbone, also induced by four heterocycles (see Figure 2c below for the patellamide structure). In one of the stable conformations, the four heterocyclic nitrogen atoms point inwards and, together with four amide nitrogen atoms, are well suited for metal ion coordination. The macrocycle also features four hydrophobic side chains, two in the expected S‐configuration and two in the inverted R‐configuration. Patellamides are found in the ascidian *Lissoclinum patella* and are produced by its obligate symbiont *P. didemni* in exceedingly large quantities [1]. Like many other secondary metabolites of cyanobacteria, they are ribosomally synthesized and post‐translationally modified peptides (RiPPs) [2]. Many such metabolites were discovered through natural product drug discovery screening programs and reported to have cytotoxic, antiviral, antimalarial, or antibacterial properties [3].

Patellamides have attracted the interest of coordination chemists for a long time [2, 4, 5, 6, 7, 8, 9, 10, 11, 12, 13, 14, 15, 16, 17]. The aqueous solution equilibria of patellamide‐Cu^2+^ systems were studied in detail by UV–vis–NIR and CD spectroscopies, ESI mass spectrometry, EPR spectroscopy and ITC [5, 8, 10], and the dicopper(II) complexes were structurally characterized by crystallography [4, 18, 19] and combinations of EPR spectroscopy with spectra simulations and structural modeling [4, 5, 19, 20, 21, 22]. The Cu^2+^ complexes have shown a wide range of catalytic activities in vitro, such as carbonic anhydrase, phosphoesterase, β‐lactamase, and glucosidase [8, 9, 23, 24]. However, so far there has not been any unambiguous evidence that the biological function of the patellamides is related to their metal binding properties.

Herein, we report experiments on biological samples of *L. patella* collected from Heron Island Reef on the Great Barrier Reef, indicating that the binding of Cu^2+^ to patellamides mediates their biological function. (i) Following an injection of Cu^2+^ solutions into the living organism, qPCR showed an increase in *patG* gene expression, and HPLC‐MS showed an increased abundance of the respective patellamide derivatives, highlighting a link between Cu^2+^ and patellamide production in the organism. (ii) XAS revealed formation of a dicopper(II) complex with a bridging carbonate originating from CO_2_. Based on known concentrations, species distributions and thermodynamic parameters, it emerges that all Cu^2+^ in the ascidians is bound to patellamides, forming carbonato‐bridged dicopper(II) complexes. (iii) Using the radioisotope ^64^Cu, we show that the presence of patellamides increases membrane permeation of Cu^2+^ into algae. These results are the first example of a biological function of a cyanobactin directly linked to metal binding.

## 2 Results and Discussion

### 2.1 Influence of Cu^2+^on Patellamide Biosynthesis

The concentration of Cu^2+^ in *L*. *Patella* is with approx. 0.5 ppm around three orders of magnitude higher than in the surrounding sea water [25], yet it is 30 to 50‐fold lower than the concentration of patellamides [26]. With a Cu^2+^ complex stability constant of log*K ≈* 6 (*K*_D_ ≈ 10^−7 ^M) [5], and p*M*_7.4_ ≈ 6 (free metal ion concentration at pH = 7.4) [27], essentially all Cu^2+^ in ascidians is expected to be coordinated to patellamides. Yet, fluorescence spectroscopy in *Prochloron* cells with a synthetic fluorescently tagged patellamide analogue is so far the only reported experiment that directly supports Cu^2+^ binding to patellamides in vivo [28].

**FIGURE 1:**
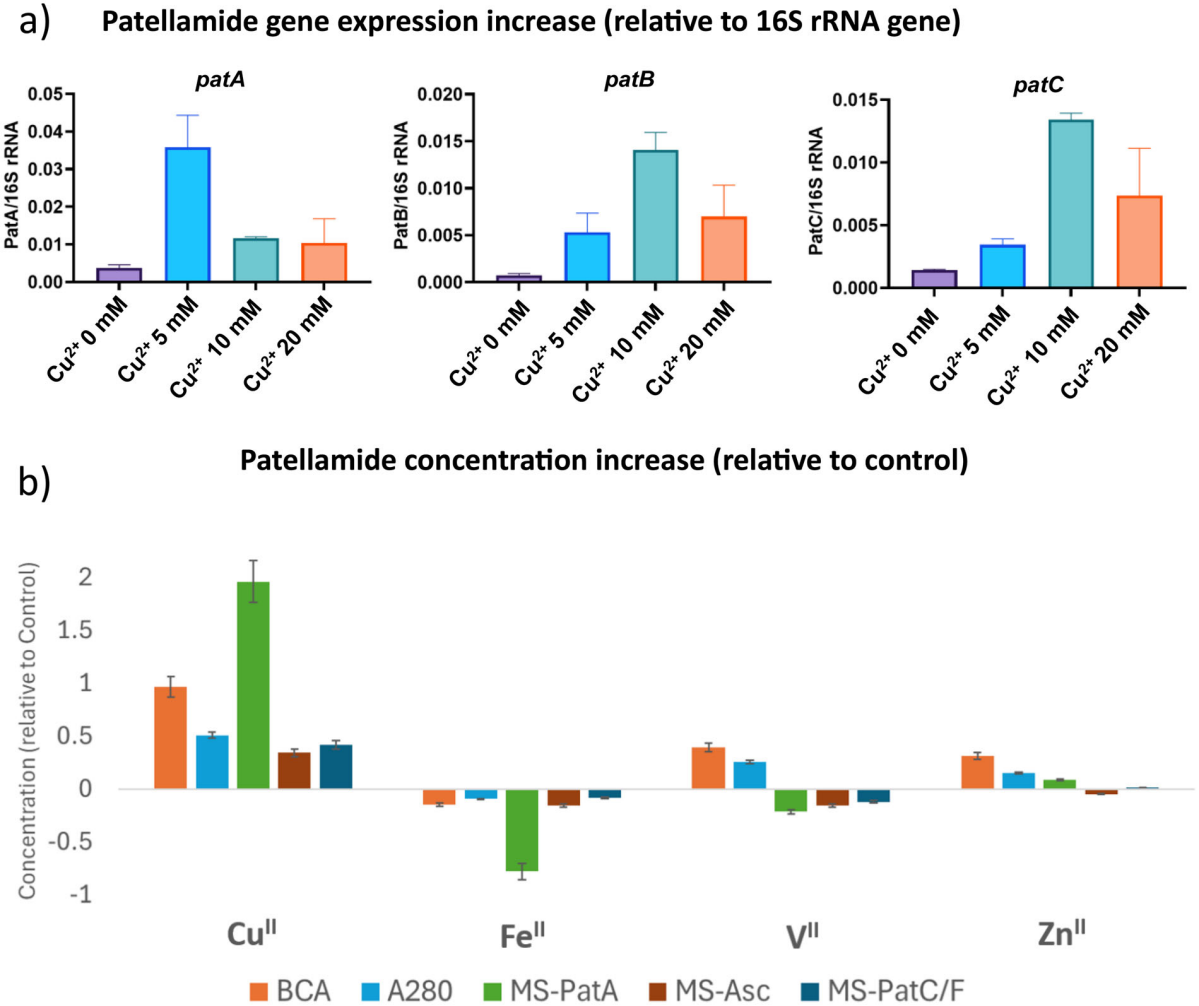
Increase in patellamide production rate and concentration, following injection of Cu^2+^ and other metal ion solutions into the ascidian cloaca. (a) Selected examples of changes in expression of patellamide‐production genes (here: patA, patB, patF, see Supporting Information for more examples and details), following the injection of 500 µL of different metal solutions (Cu^2+^ 5, 10, 20 mM; the data for other metal ions such as Fe^2+^ 10 mM, Zn^2+^ 10 mM, V^2+^ 10 mM can be found in the Supporting Information). (b) Relative concentration changes of total protein (determined by BCA assay, PHERAStar BMG absorption at λ = 280 nm) and patellamides (determined by HPLC‐MS for patellamides A, C/F and ascidiacyclamide, note that patellamides C and F cannot be distinguished by MS because they have the same exact molecular weight) after injection of different metal solutions (Cu^2+^ 10 mM, Fe^2+^ 10 mM, Zn^2+^ 10 mM, and V^2+^ 10 mM).

The observation that the Cu^2+^ concentration in the ascidian *L. patella* is significantly higher than in the surrounding sea water, and that Cu^2+^ is coordinated to patellamides, indicates that Cu^2+^ binding might be important for the biological role of these peptides. To determine whether the patellamide production is related to the Cu^2+^ concentration inside the organism, we injected Cu^2+^ into the cloacal cavities of ascidians in vivo (see Figures 1, S2, S3). Patellamide concentrations were determined by LC‐MS and the total protein content by a BCA assay (see Supporting Information for details). After 24 h, the concentration of patellamides was found to have increased significantly in all parts of the ascidians injected with Cu^2+^ (approx. 2‐fold compared to blanks).

Subsequent evaluation of the expression rates of the genes required for the biosynthesis of patellamides (*patA*‐*patG*, with *patE* encoding the precursor peptide) showed that, all gene expression rates (relative to the 16S rRNA gene) were notably increased compared to control samples of the ascidian that were only injected with water (see Figure 1a). No concentration dependence was found in either the patellamide quantification or gene expression rates. However, it is likely that at the concentrations used, the maximum production rate was already reached and that a concentration dependence might, therefore only be noticeable at lower concentrations. Injection with Zn^2+^, Fe^2+^, or V^2+^ salts instead of Cu^2+^ resulted in no substantial increase in general gene expression or patellamide concentration, although the expression rate of selected patellamide genes was slightly increased (see Figures S6, S7). The reason for this effect is unclear, but it might indicate that the pathways regulating patellamide expression are to a certain extent influenced by other metal ions as well as Cu^2+^.

The results show that Cu^2+^ in the ascidians stimulates patellamide production by the symbiont *P. didemni*. Together with the observation that all Cu^2+^ in the ascidians is coordinated to patellamides, this indicates that the biological function of the patellamides is likely related to their Cu^2+^ complexes.

### 2.2 EXAFS Studies of Biological Samples

**FIGURE 2:**
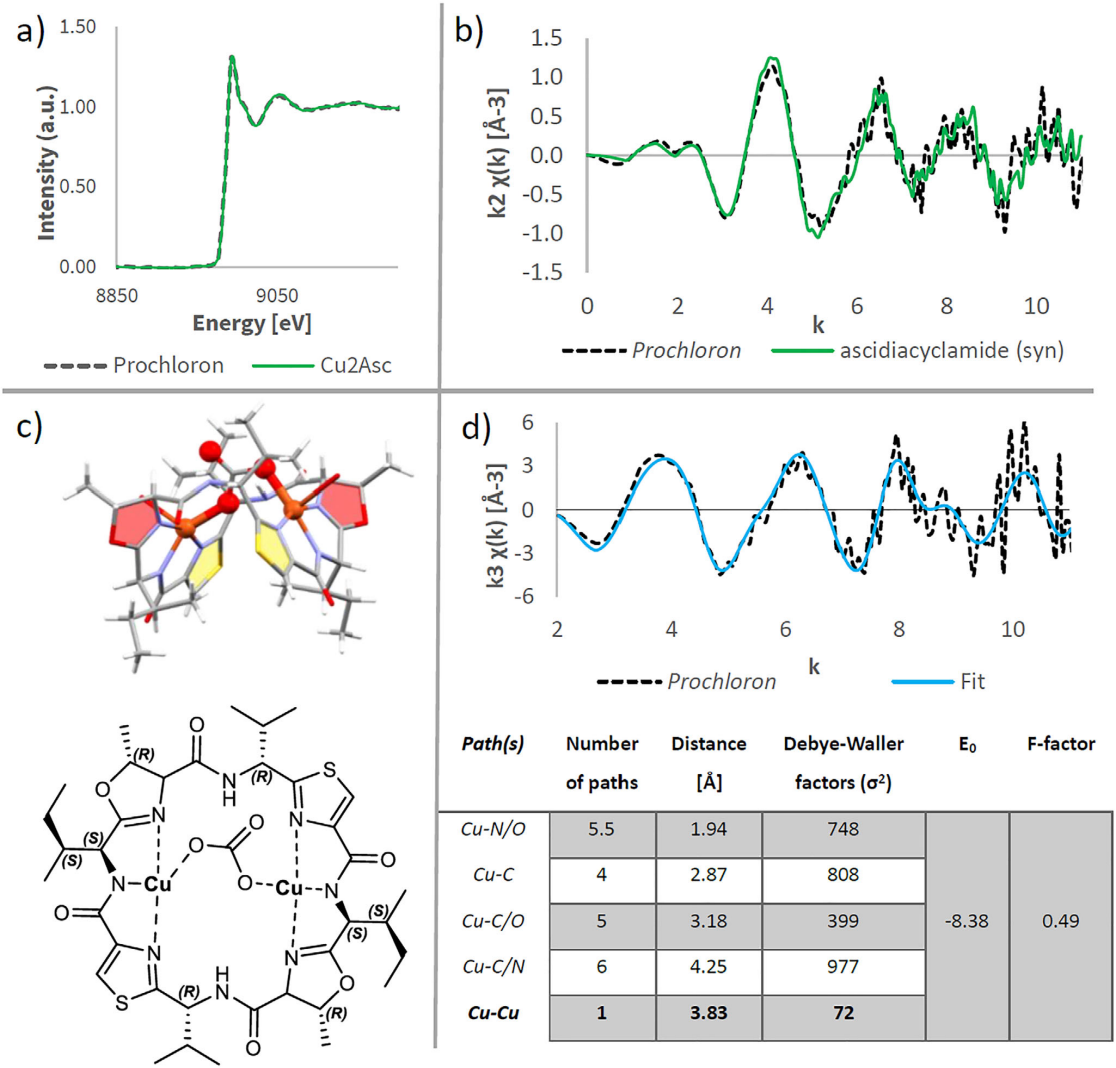
XAS data: (a) Overlay of the XAS data of the biological (green) and the synthetic Cu^2+^ complex (black). (b) Overlay of the EXAFS region of the XAS data of the biological sample (black) and the synthetic Cu^II^_2_‐ascidiacyclamide complex (green); the EXAFS is shown with a k‐weighting of two to enhance high‐*k* oscillations while maintaining visibility of low‐k features. (c) X‐ray crystal structure [4] of the Cu^II^_2_‐ascidiacyclamide complex (structural drawing at the bottom). (d) Overlay of the experimental EXAFS spectrum (black) of the biological sample and the calculated spectrum (blue) based on the x‐ray structural data of the Cu^II^_2_‐ascidiacyclamide complex; fitting parameters are tabulated. The EXAFS and fit are shown with a *k*‐weighting of three, and the fit was performed in the range *k* = 2–11 Å^−1^ (see Supporting Information for additional EXAFS data, Figures S20–S25).

Unambiguous support for Cu^2+^ binding and structural properties of Cu^2+^‐patellamide species in vivo by UV‐vis‐NIR, CD and EPR spectroscopy is hampered by low resolution, low‐intensity transitions, and intense background signals from other species present that cover the weaker signals of the Cu^2+^ patellamide complexes, such as “impurities” of the naturally more abundant and more intense signal of Mn^2+^ species in the case of EPR spectroscopy. This makes electronic and magnetic resonance spectroscopies unsuitable for the identification and accurate analysis of Cu^2+^‐patellamide species in vivo. Therefore, x‐ray absorption spectroscopy (XAS) was used to analyze both the biological samples and Cu^2+^ complexes of synthetic ascidiacyclamide [29], because XAS enables the detection of specific metal complexes in vivo / ex vivo, if the concentration ratio of the metal complex of interest relative to free metal ions and complexes with other ligands is large enough [30]. The entire XAS and EXAFS regions of the samples with biological and synthetic material show high similarity (see, Figures 2 S20–S25). Because the concentration of Cu^2+^ in biological samples is much lower than in samples of synthetic patellamides [25], the noise level in the spectra of biological samples is much higher. To optimize the signal, the biological samples were measured multiple times at different positions, and the data was averaged. While in the low‐*k* region of the EXAFS (corresponding to short distances) the spectra of biological and synthetic samples are nearly identical, the larger distances show small deviations likely due to increasing noise or the presence of at least five different patellamide derivatives. While the coordination sphere around the absorbing Cu^2+^ should be close to identical for the various patellamides, at *k* values corresponding to distances larger than ∼3.5Å the differences in the side chains may impact the spectrum increasingly.

The fit of the EXAFS data is based on reported x‐ray data of the carbonato‐bridged dinuclear Cu^2+^ complex of ascidiacyclamide (see Figure 2c) [4]. These structural parameters are almost identical to those of a solution structure refined by a combination of EPR spectroscopy, spectra simulations and structure optimization based on force field and DFT calculations [8, 20, 21, 22]. The similarities between the data collected from the biological and synthetic samples (Figure 2b), as well as between the biological sample and the fit to the experimental structure (Figure 2d), suggest that most of the Cu^2+^ in the ascidian cloaca and the cyanobacteria is bound to patellamides. Importantly, the XAS experiments show that the structures of these complexes agree with those determined in (frozen) solution by EPR spectroscopy and in crystals by x‐ray diffraction (Figure 2d). As the fit to the crystal and solution structures indicates, these complexes are bridged by carbonate. Attempting to fit the spectra to an experimental or DFT‐optimized structure without a bridging carbonate leads to structures that show considerably larger Cu···Cu distances (≈5.2 Å vs. ≈3.8 Å) [5, 8, 9, 23, 24] and produce significantly worse fits (see Figure S25 for details).

In summary, XAS shows that Cu^2+^ in the ascidians is coordinated to patellamides, forming dicopper(II) complexes with structures identical to the carbonato‐bridged dicopper(II) complexes that have been determined experimentally with synthetic patellamide analogues.

### 2.3 Carbonic Anhydrase Activity

Dicopper(II)‐patellamide complexes are known to be very efficient carbonic anhydrases. In fact, these dicopper(II) complexes catalyze the hydration of CO_2_ faster than any other known biomimetic carbonic anhydrase model [8, 24, 31]—note that the enzymes and typical model systems generally are mononuclear Zn^2+^ complexes. However, a recent computational analysis revealed that the rate limiting step in the catalytic carbonic anhydrase cycle of the dicopper(II)‐patellamides is not the attack of the OH^−^ nucleophile, coordinated to one of the Cu^2+^ centers, at the carbon of CO_2_, coordinated to the other Cu^2+^ center; it rather is the release of the carbonate product, bridging the two Cu^2+^ centers in the final intermediate. Importantly, at low pH the carbonate bridge is protonated and HCO_3_^−^ release has a lower energy barrier, while at higher pH release of the bridging CO_3_^2^^−^ has an exceedingly large barrier, indicating product inhibition of catalytic CO_2_ hydration at high pH [31]. This is in accord with experiments (stopped‐flow kinetics), where at pH>8 there is a sharp decrease of the reaction rate [24]. The pH value in the cloaca of ascidians is known to be ∼10 during the day and ∼7 at night [2, 32]. Therefore, catalysis is inhibited during the day, so it is unlikely that carbonic anhydrase activity is the natural function of the patellamides. This is supported by the fact that the patellamides are produced in much larger amounts than expected for catalysts (*vide supra*).

It appears that essentially all Cu^2+^ in the cloaca of the *Lissoclinum* ascidians is coordinated to patellamides in dicopper(II) complexes with a bridging carbonate. This suggests that a possible biological function of the patellamides is to transport carbonate to the ascidians’ photosynthetic symbiont *Prochloron*, rather than acting as a carbonic anhydrase.

### 2.4 CO_2_ Transport

From exhaustive studies of the aqueous patellamide/Cu^2+^ system it emerges that there is cooperative binding of two Cu^2+^ centers to a patellamide macrocycle, and that under ambient conditions, a bicarbonate or carbonate is bridging the two metal centers (*vide supra*) [5, 8]. This was already observed in early work, when attempting to obtain nitrate‐bridged complexes [4, 33]. An important observation is that most carbon‐fixating cyanobacteria have an intracellular pH value of eight or above to help prevent CO_2_ leakage [34], and these organisms typically use carbonate transporter molecules such as NdhD3‐F3 and low‐CO_2_ inducible bicarbonate transporters sbtA and CmpA‐D [35]. *P. didemni* lacks these transporters [36] and has a lower intracellular pH (*vide infra*) [32].

Preliminary experiments on the bioactivity of patellamides and their Cu^2+^ complexes, using the algae *Nannochloropsis* spp., demonstrate that (i) patellamides alone have no significant effect on algal vitality, (ii) low Cu^2+^ concentrations (∼1 ppm) combined with patellamides significantly increase the photosynthesis rate, and (iii) higher concentrations lead to increased Cu^2+^ toxicity (see Figures S4, S5, S34–S36). To evaluate this, *Nannochloropsis* spp. cultures were treated with different concentrations of Cu^2+^, patellamides, and a combination of both. Algal vitality was monitored regularly using a PAM chlorophyll fluorometer under both dark‐ and light‐adapted conditions (see Supporting Information). The rapid changes observed suggest that the patellamide‐copper(II) complexes are membrane permeable.

**FIGURE 3:**
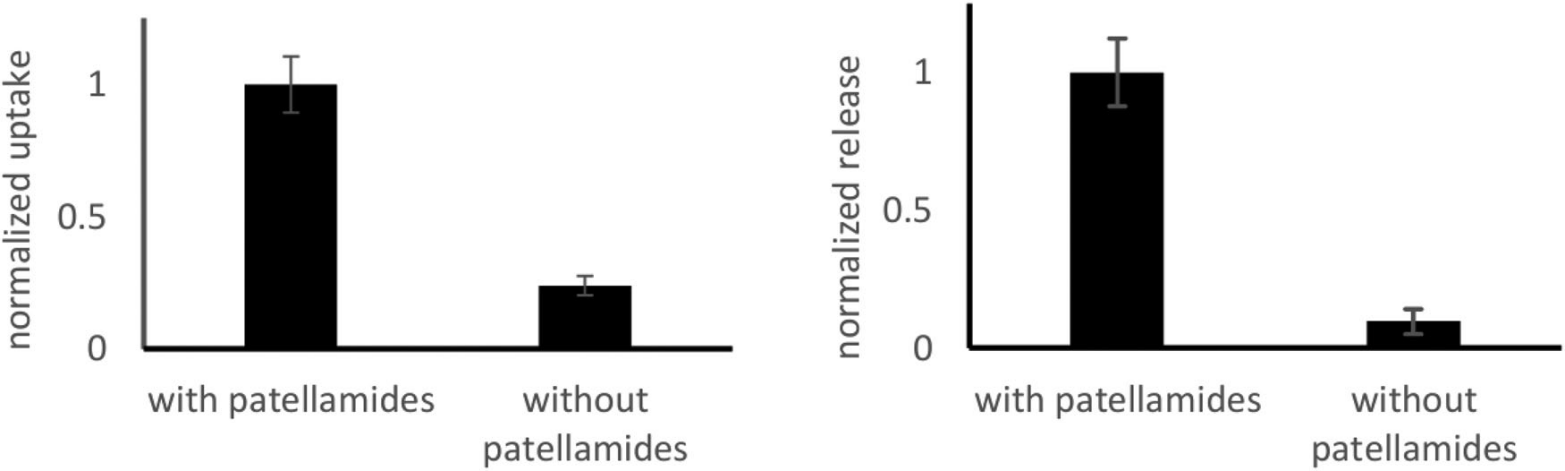
Count rates (CPM) in cultures of *Nannochloropsis oculata* used to estimate ^64^Cu^2+^ uptake and release with and without patellamides; it emerges that both uptake and release are strongly influenced by the presence of patellamides (Table S3).

This is an important observation in the context of CO_2_ transport in the ascidian‐*Prochloron* symbiosis and was therefore further tested with radiotracer experiments using ^64^Cu^2+^ (β^+^, *t*_1/2 _= 12.7 h). To evaluate the ^64^Cu^2+^ uptake and release with and without patellamides, *Nannochloropsis oculata* was used as a model system. This was necessary because, when evaluating the uptake for *P. didemni*, the healthy cyanobacteria release such high amounts of patellamide that a negative control is not possible. Algal cultures in artificial sea water were incubated for 2 h with ^64^Cu^2+^, with or without added patellamide. These mixtures were then centrifuged and the radiation count rates in counts per minute (CPM) associated with both the supernatant and the pellet were determined. The radioactive algae were then resuspended in artificial sea water, incubated with or without patellamides, centrifuged again, and the count rates of the supernatant and pellet were determined. The results show that the cultures of *Nannochloropsis* spp. take up and release significantly higher amounts of Cu^2+^ when patellamides are added (Figure 3 and Table S3). This is especially noteworthy, since *Nannochloropsis* spp. is an alga with a rigid cell wall and is not expected to have evolved any active uptake mechanism for copper(II)‐patellamide complexes. Therefore, this supports the hypothesis that the carbonato‐bridged dicopper(II) complexes of the patellamides can penetrate the membranes of the cyanobacterium *Prochloron*.

**FIGURE 4:**
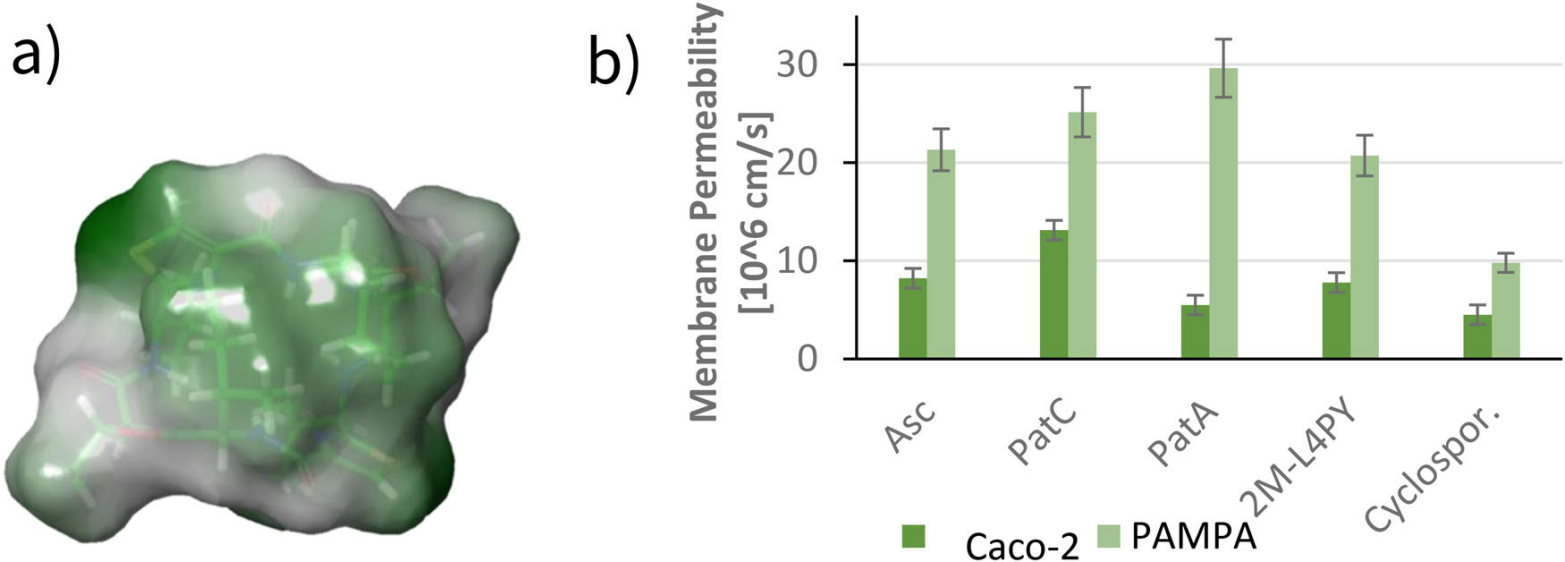
(a) Hydrophobic surface area map (green: hydrophobic, white: neutral, blue/red: polar) of the patellamides, represented by ascidiacyclamide (structural data from the dicopper(II) complex POHKOM [4] plotted after removing metal ions), showing a large hydrophobic patch extending over most of the molecular surface. (b) PAMPA and Caco‐2 membrane permeability data for three patellamides (patellamide A, patellamide C, ascidiacyclamide) compared to known permeable compounds, 2M‐L4PY (cyc‐LLLLPY [39]) and cyclosporine. All compounds were measured concurrently; values for cyclosporine represent the highest reported literature values [40].

To further evaluate the membrane permeability of patellamides and their Cu^2+^ complexes, a series of measurements were performed using the well‐established PAMPA (parallel artificial membrane permeability) and Caco‐2 (human colorectal adenocarcinoma cell monolayer) assays. The results in Figure 4b (see Supporting Information for details) show that patellamides cross both artificial membranes and cell monolayers with extremely high permeabilities (*P*_app,PAMPA_ = 29.6 × 10^−6^ cm/s [patellamide A]; *P*_app,Caco‐2 _= 13.12 × 10^−6 ^cm/s [patellamide C]; see Table S4 for tabulated values). For patellamide C, the value *P*_app,PAMPA _= 25.1 × 10^−6 ^cm/s agrees with previous findings of high passive permeability (*P*_app,PAMPA _= 18.8 × 10^−6 ^cm/s) [37]. Notably, while all three derivatives show high permeability, *P*_app_ is greater for patellamide A than for ascidiacyclamide, where the only structural difference is a methyl group on one oxazoline heterocycle. Furthermore, the cell monolayer permeability *P*_app_ of patellamide C in Caco‐2 is almost double that of ascidiacyclamide, suggesting that the nature of hydrophobic sidechains plays an important role in cell membrane permeability. This high membrane permeability is likely due to the observed relationship between hydrophobic surface patch size and permeability [38] and removal of exposed amide protons (Figure 4a) [39]. While the corresponding dicopper(II) complexes did not show significantly different permeability, there is a substantial increase in Cu^2+^ transport, as shown by experiments with ^64^Cu^2+^. This suggests that the biological function of patellamides is to utilize this high membrane permeability to transport Cu^2+^ and carbonate into the cyanobacteria.

The conclusion so far is that two Cu^2+^ ions are coordinated to patellamides in the atrial cavity of the ascidians and that CO_2_ is efficiently hydrated by this species, leading to a stable carbonato‐bridged complex at the relatively high pH in the ascidian. This metal complex can cross the membrane into the cyanobacterial symbiont *Prochloron*. We have measured the intracellular pH in *Prochloron* using the pH‐dependent ratiometric fluorescent dye BCECF/AM (Figures S8–S11, Table S2): at the observed pH = 6.9 ± 0.1, based on experimental evidence [24] and computational modeling [31], HCO_3_^−^ is released immediately and available for photosynthesis.

As previously noted, the concentration of patellamides in didemnid ascidians is high (∼ 0.5 mM), and generally, all Cu^2+^ (∼ 0.05 mM) is bound by these compounds. The high concentration of copper(II)‐patellamide complexes suggests a role in CO_2_ transport, rather than any catalytic (enzymatic) process mediated by patellamides. It appears that the photosynthetic activity of *Prochloron* is limited by the amount of Cu^2+^ available in the ascidian, and the energy intensive production of patellamides by the cyanobacterium is directly linked to this Cu^2+^ concentration. Furthermore, this excess of patellamides protects *Prochloron* cells by preventing cytotoxicity through accumulation of free copper.

## 3 Conclusion

This is an important development in chemical biology and marine science. It shifts patellamides from the category of being secondary metabolites with unknown function to functional mediators of symbiosis. The research links structural chemistry (carbonato‐bridged dicopper(II)‐patellamide) directly to biological activity within the host/symbiont environment. The experimental data presented here, specifically the observed increase in patellamide production rate and concentration following injection of aqueous Cu^2+^ salts, along with XAS data suggesting the presence of carbonato‐bridged dicopper(II)‐patellamide complexes in vivo (with structures identical to synthetic analogues), lead to the conclusion that patellamides are produced by *P. didemni* to bind Cu^2+^ to enable CO_2_ fixation and transport. This Cu^2+^ binding is supported by recent studies demonstrating that reversible Cu^2+^ complexation occurs in vivo [28]. An interesting observation is that these carbonato‐bridged dicopper(II) complexes can penetrate the membranes of different organisms and positively impact the vitality of algae. Given the known high carbonic anhydrase activity of patellamides [24] and the lack of carbonate transporters in *P. didemni* [32], a hypothesis that might explain all observations is that the patellamides serve as Cu^2+^‐based carbonate transporters. Importantly, this can explain the large amounts of patellamides produced by *P. didemni* [41], as the investment in biosynthesis is justified if the increased carbonate availability for photosynthesis outweighs the cost. A metal‐related function of the patellamides is the first example of this kind for a cyanobactin, and the hypothesized carbon fixation mechanism is a new example of a special carbon fixation strategy, developed by this unusual photosymbiotic cyanobacterium inhabiting the cloacal cavity of a sessile colonial ascidian. This might be the first of many examples of unexpected biological functions for molecules previously studied only for their pharmacological properties.

## Author Contributions

**Philipp Baur**: conceptualization, data curation, investigation, writing – original draft, validation, formal analysis, visualization. **Timothy A. Hill**: investigation, validation, formal analysis, writing – original draft. **Kai‐Chen Wu**: investigation, validation, formal analysis. **Junxian Lim**: investigation, validation, formal analysis. **Brett M. Paterson**: investigation, validation, formal analysis, writing – review & editing. **Gunasekaran Velmurugan**: investigation, validation, formal analysis. **Bernard M. Degnan**: data curation, resources, writing – review & editing. **David P. Fairlie**: supervision, funding acquisition, resources, writing – review & editing. **Peter Comba**: conceptualization, data curation, supervision, funding acquisition, project administration, writing – original draft, writing – review & editing, formal analysis.

## Conflicts of Interest

There is no competing interest to declare.

## Supporting information




**Supporting File**: This includes the materials and methods section, a detailed description of the field experiments and sample collection, metal‐injection experiments, XAS/EXAFS experiments, ^64^Cu^2+^ experiments, determination of intracellular pH values, bioactivity measurements and cell membrane permeability assays, as well as a compilation of the corresponding data in tables and figures.

## Data Availability

The data that supports the findings of this study is available in the supplementary material of this article.
