# Supplementary material for: Experimental Evidence for a Metal‐Related Function of a Cyanobactin
Source: Angew Chem Int Ed Engl. 2026 May 2;65(25):e8567749. doi: 10.1002/anie.8567749 (PMC13266932; doi:10.1002/anie.8567749)
Supplement: Supplementary file 1 — Supporting File: This includes the materials and methods section, a detailed description of the field experiments and sample collection, metal‐injection experiments, XAS/EXAFS experiments, 64Cu2+ experiments, determination of intracellular pH values, bioactivity measurements and cell membrane permeability assays, as well as a compilation of the corresponding data in tables and figures. [file ANIE-65-e8567749-s001.docx]

# **Supporting Information^[[1]](#footnote-1)^**

**Table of Contents**

Materials and methods 2

*Mass spectrometry* 2

*Determination of intracellular pH* 2

*Computational methods* 2

*Field experiments and sample collection* 3

*Metal ion injection experiments* 4

*RNA extraction and quantitative RT-PCR* 6

*Impact on patellamides and copper on algae* 7

*X-ray absorption spectroscopy* 7

*^64^Cu uptake experiments* 8

*Determination of intracellular pH* 9

*Bioactivity of patellamides and copper* 11

*Parallel artificial membrane permeability assays (PAMPA)* 15

*Caco-2 cell membrane permeability assay* 15

Supplementary data 17

*qPCR* 17

*^64^Cu experiments: raw data* 19

*Intracellular pH value determination* 20

*BCA and PHERAstar measurements* 23

*HPLC-MS data (injection of metal ion solutions into the ascidians* 24

*XAS data* 25

*HPLC-MS data*  29

*Patellamide membrane permeability data (PAMPA and Caco-2)* 33

*Patellamide bioactivity towards Nannochloropsis spp.* 34

References 36

## **Materials and Methods**

**Mass spectrometry**

High-Resolution-Triple-TOF mass spectra were recorded using a Sciex TripleTOF+ 5600 and a TripleTOF+ 6600 by Alun Jones at the mass spectrometry facility at the Institute of Molecular Bioscience of the University of Queensland, Brisbane, Australia. These Triple-TOF files were interpreted using the Sciex OS package as well as the Open MS package.

**Determination of intracellular pH values**

The freshly harvested cyanobacteria were directly incubated with BCECF/AM, initially the ideal incubation conditions were tested by first incubating for 30 minutes at room temperature and by subjecting the cyanobacteria to an acidic shock (1 minute in 1M HCl), followed by 30 minutes incubation with BCECF/AM – and comparing different concentrations of BCECF/AM (25 µM, 10 µM, 5 µM). As the cyanobacteria did not survive the acid treatment, cultures were directly incubated with BCECF/AM. While all concentrations gave a measurable signal, 10 µM was considered the ideal concentration.

All plate reader measurements were performed at the IMB of the University of Queensland, Brisbane, in 8-fold repetition on a 96-well plate. The measurements were performed using a Tecan Spark Multimode plate reader. Confocal microscopy was performed using the Zeiss Axiovert 200 Inverted Microscope Stand equipped with a LSM 710 Meta Confocal Scanner at the IMB microscopy facility.

**Computational Methods**

Gaussian 09^[1]^ with B3LYP-D3 (LACVP basis set) was used for geometry optimizations and ef2-TZVP for single point calculations.^[2]^ The solvation energies were computed by using the continuous polarizable continuum (CPCM) solvation model, with methanol as solvent. All reported energies are B3LYP-D3 solvent-phase energies incorporating free energy corrections at 298.15 K, unless otherwise mentioned.

## **Field Experiments and Sample Collection**

Intact specimens of *Lissoclinum patella* with sizes of 20-160 cm^2^ and thicknesses of 5-15 mm were collected in June-July 2022 and August 2023, on the Heron Reef at depths mostly between 0.5 m and 6 m on Heron Island (near Blue Pools Area, 23°26'04.4"S 151°55'21.1"E, Libby’s Lair Area 23°43’40.4”S, 151°93’41.0”E). All specimens were transported in fresh seawater as quickly as possible to the Heron Island Research Station, where they were kept in shaded outdoor aquaria (less than 200-300 µmol Photons/m^2^xsec) with continuous supply of fresh reef water. The collected animals were handled using sterile or sterilized equipment. All samples collected were kept at dry ice temperature or below until they were processed.

**Figure S1.** Map of Heron Reef from the top, primary collection (depths between 1m and 6m) area marked in orange. (Credit: CSIRO / ©ESA)


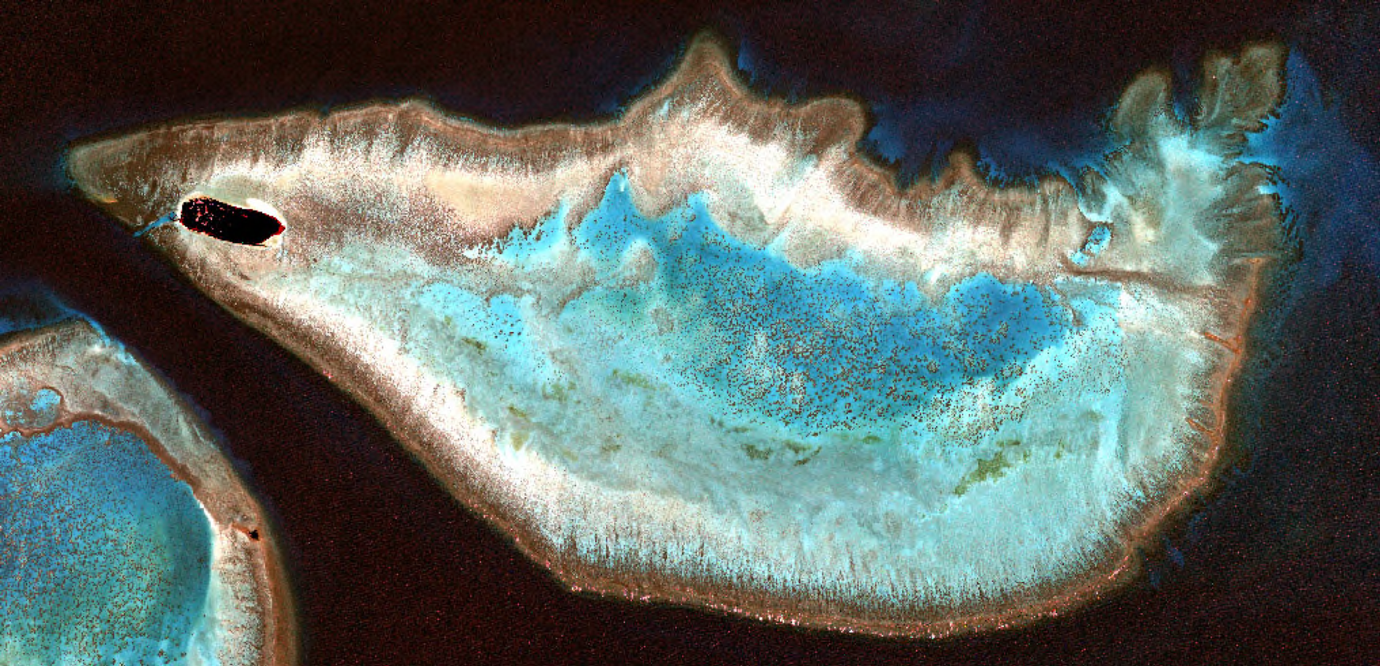


For the cyanobacterial samples, the cyanobacteria and the cloacal liquid were collected from the specimen by cutting the ascidian longitudinally with slight mechanical pressure, which causes the liquid with the cyanobacteria to flow out. For the homogenized samples used in the XAS experiments, a glass/Teflon Potter Elvehjem homogenizer was used. To obtain only the water from the cloaca, the cyanobacteria were centrifuged off; for the filtered samples, a syringe filter (0.45 µm CA) was used.

In the sample “copper added” (specimen 3), a solution of 3 x 2 mL 14.2 mM Cu(SO_4_) 6 H_2_O was injected to one section of the living species, the other section was treated similarly with fresh water. The specimen was then kept in the aquarium before preparing it as described above (freezing, cutting, separating cloaca/Prochloron, homogenizing etc.).

For the measurements requiring living cyanobacteria e.g., for as the determination of the intracellular pH, it was necessary to transport living ascidians to the laboratories in Brisbane. As the cyanobacteria typically start to die within 1-3 days outside of their host, the properties might not be representative of healthy cells even though they might still be alive. Therefore, the cyanobacteria were transported inside healthy ascidian hosts. The ascidians were transported in plastic bags filled with fresh reef water inside an esky box, the water regularly being replaced by fresh reef water, separately transported.

For samples of the water within the cloaca, either a syringe or disposable pipette were used – or the organism was cut longitudinally, and the cloaca content carefully squeezed out, and subsequently any larger particles and cells were filtered off, using cellulose acetate filters. For measurements involving the DCM extract, the sea squirt was cut into small pieces, homogenized using a glass Teflon homogenizer and the mixture (without the outer mantle tissue) was stirred with an equal volume of DCM for 12 hours in a covered Erlenmeyer flask and the organic phase was isolated using a separating funnel. For samples using cyanobacteria, the sea squirt was cut longitudinally, and the cyanobacteria and water were gently squeezed out.

All samples were immediately stored in a -80 °C freezer and have continuously been transported in dry ice-cooled containers.

**Metal ion injection Experiments**

For the measurements related to the impact of the injection of different metals and metal concentrations into the living animal (cloaca), 3 healthy colonies were chosen to be injected with 5mL of different metal solutions, prepared from freshly made 10mM (Cu/Fe/Zn) or 1mM (V) stock solutions of the metal salts CuSO_4_, FeSO_4_, Zn(Ac)_2_, V_2_O_5_ in purified (filtered and denatured) reef water:

Specimen 1 (Cu): Cu^II^ – 5mL of 0 (Water), 5 mM, 10 mM and 20mM CuSO_4_ solutions

Specimen 2 (Fe): water, 10mM Cu^II^ Fe^II^ Zn^II^

Specimen 3 (V): water, 10mM Cu^II^ V^II^ Zn^II^


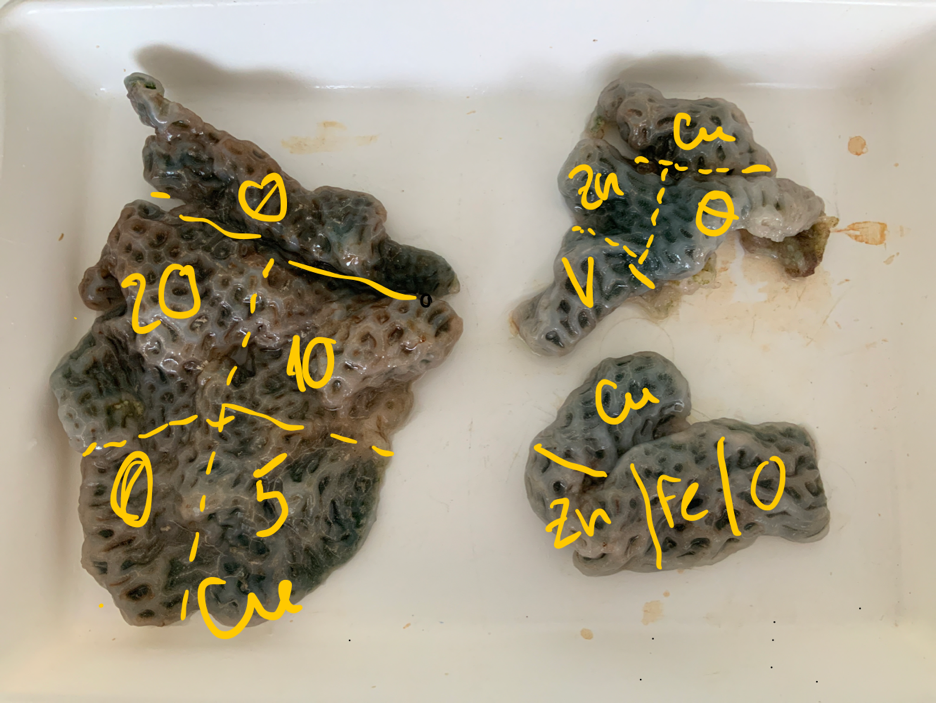
The colonies were injected using a hypodermic needle into of previously chosen areas with at least 2 cm of distance between the injected areas to minimize the probability of cloacal cavities being interconnected. The three specimens were taken from their aquaria for injection midday (11:30-12:30), injected into the areas shown in Figure S2 and after the injections have been kept at the same terrace in triple-contained separate aquaria with regularly exchanged water (Figure S3).

**Figure S2**. The three colonies used for the injection experiments with the areas of injection highlighted.

**
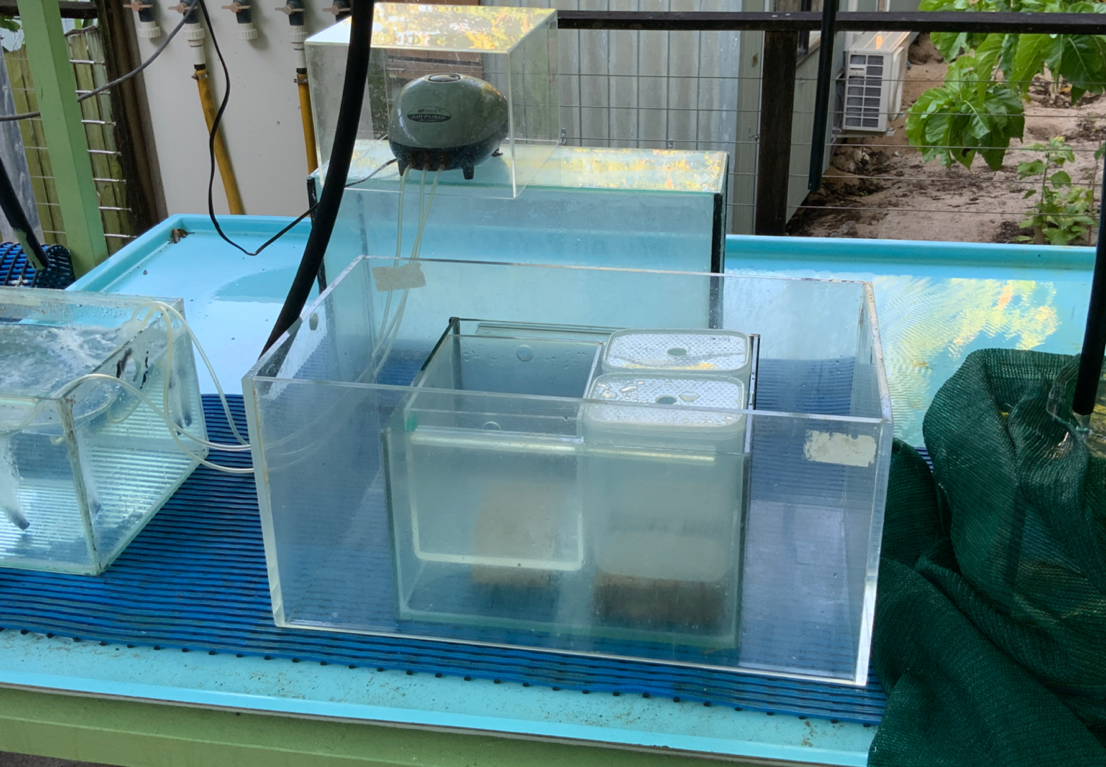
**

**Figure S3**. The triple-containment aquarium setup used. The aquarium pump shown was used to aerate the water during the experiments.

After 24 hours, the colonies were taken back to the lab, dried off on outside and subsequently cut along the lines shown in Figure S2, discarding the ≈1-2 cm of tissue in between. The separated samples were then cut into smaller pieces of colony and cut longitudally along the surface to extract the cyanobacteria and the surrounding cloacal water until no visible liquid and cyanobacteria remained in the mantle tissue.

Of the resulting samples, 2 mL were filled into Eppendorf tubes and directly frozen at -80 °C, 2mL were centrifuged, the supernatant filled into Eppendorf tubes and frozen, the pelleted cyanobacteria were resuspended in 1mL RNAlater, kept overnight in the refrigerator at 4 °C overnight and frozen at -80 °C subsequently.

The frozen samples were later filtered and used for the evaluation of patellamide/protein content by LC-MS, BCF assay and PHERAstar BMG_A280nm (absorption-based protein quantification assay).

**RNA extraction and quantitative RT-PCR**

For the qPCR experiments, both samples directly frozen after harvesting and frozen after storage in RNAlater were used and compared. As only the samples that have been frozen with RNAlater showed sufficient amounts of RNA for interpretation, the results of the directly frozen samples are omitted.

For RNA extraction, samples were homogenized with 0.9-2.0mm stainless steel beads (Next Advance) at 4 °C in 500 µL TRIsure™ (Bioline) in a Bullet Blender® tissue homogeniser (Next Advance). Samples were then removed from beads and added 100 µL chloroform and vortexed vigorously. After a 5-minute room temperature incubation, samples were centrifuged at 12000 rpm for 10 minutes at 4 °C, and the clear supernatant layer was collected for RNA isolation. RNA isolation was conducted with ISOLATE II RNA Mini Kit (Bioline) according to manufacturer’s protocol. cDNA generation was carried out on a VeritiTM 96 well Thermal Cycler (Applied Biosystems). Real-time PCR was performed using ViiA 7 Real-Time PCR System (Applied Biosystems). Primer sequences are designed using PrimerQuestTM Tool (INTEGRATED DNA TECHNOLOGIES) and described in Table S1.

**Table S1.** Primer sequences for target genes

| Gene | Forward | Reverse |
| --- | --- | --- |
| PatA | TCTCTACGGTTGGAAGGTTAATG | CATCCAAGGTTTGGCGAATG |
| PatB | GAGAATGGCCGTCTTAAAGATTTG | GTGCATCACTTGGTTGAAACTG |
| PatC | CAACCCTACCGCACAATCTAA | GTAGGCGCACCAATAAGAGTAG |
| PatD | CCCACTTCCACGTTGAGATAA | GAAAGGCAGAATTTGGCAGTAG |
| PatE | CTGAACTGTCTGAGGAAGCTCT | GCGCAAACACTGATGCAAAC |
| PatF | CAATTTGTCCGAACCCATCAAG | GCAAGAGGATTCAACAGAACAAG |
| PatG | CAACCCTACCGCACAATCTAA | GTAGGCGCACCAATAAGAGTAG |
| 16s rRNA | CGGAGGATGCAAGCGTTAT | ACTGCCTTTCCGGAGTTTAG |

**Impact of patellamides and copper on algae**

For the observations concerning the impact of the addition of copper, patellamides or both, the easily available and commonly used algae of the genus *Nannochloropsis spp.* were used. For the first set of measurements with the higher copper concentrations, *Nannochloropsis gaditana* and *Nannochloropsis oculata* were obtained from the lab of Prof. Annika Guse at the Center of Organismal studies, Universität Heidelberg. Due to lack of access to the former source, for the second series of measurements *Nannochloropsis salina* produced by NYOS Aquatics was purchased. The algae were stored in a darkened room under a lamp simulating the light composition at a water depth of 1 meter (SolarStinger LED SunStrip Marine).

For all PAM measurements, a Walz MINI-PAM-II chlorophyll fluorometer was used. The data analysis has been performed using Microsoft Excel 365.

In all measurements, 2 mL of the algae culture were filled into heat-sterilized screw-cap glasses. To these cultures, either 100 µL of a patellamide-containing solution (1.32 mM in DMSO for all synthetically produced patellamides, or the denatured, filtered aqueous cloaca extract), a copper solution (100 µL of 15 mM CuCl_2_x2H_2_O in the first series of measurements, 25 µL of 1.2 mM CuCl_2_ in the second series of measurements) or both were added. In case of the control cultures 100 µL of the solvent, DMSO or H_2_O, was added instead.

All algae samples were measured simultaneously in regular intervals, always once light - and once dark-adapted.

## **X-ray Absorption Spectroscopy**

Initial X-ray absorption spectroscopy measurements were performed at the mini-undulator beamline P65 of DESY Hamburg in April 2021 after introduction by the beamline-scientist Edmund Welter. The samples were measured in self-designed sample holders made from PEEK, POM and polyimide and made by the fine mechanic workshop of the department of inorganic chemistry at Heidelberg University. The sample environment used a He-cooled cryostat and a precision motorized sample holder. The fluorescence signal was measured using a PIPS-diode detector (due to the low concentration of copper in the samples an HPGe detector was asked for in the proposal, but it was not available at the time).

The main measurements were performed at the bending magnet beamline SuperXAS at the Paul Scherer Institute in Villigen, Switzerland in January and August of 2022 and were set up by the beamline scientist Grigori Smoletsev. The samples were mostly measured in thin-walled (10µm) quartz glass capillaries made by Hampton Research, the freeze-dried biological samples were measured in 0.3 mm wall thickness Kapton tube capillaries made by DuPont. The sample environment used a liquid nitrogen-cooled cryojet (100K) and a sample holder for 1-3 capillaries mounted to a precision-engine block. The fluorescence signal was measured using a 5-element silicon drift detector.

After determining the optimal measurement coordinates of the sample table engines, ensuring correct alignment of the sample within the beam. For all samples, initially the photodamaging was evaluated, by repeatedly measuring 5-minute XANES spectra at the same spot and looking for photodamage-related changes, to choose a measurement time at which the impact of photoreduction on the spectrum is minimal. For all samples, measurement times of 16 min and 55 seconds for EXAFS measurements and 5 minutes for XANES were chosen at PSI (at DESY, 20 minutes). For each EXAFS measurement, a successive XANES measurement was performed at the same spot, to be able to estimate whether the sample might have been photodamaged at this spot. For the more concentrated samples such as frozen liquid solutions of the synthetically produced ligands the quartz capillaries were used and measured at 4-6 positions. For the biological samples, Kapton capillaries leading to a stronger fluorescence signal were used, measuring as many points as possible (considering the filling height of the capillary, the beam size and a buffer around each spot), which in most cases were 12-16 spots. The python scripts for the measurement were adapted from the ones provided by the beamline.

The measured spectra were initially processed using Athena from the Demeter Package^[3]^ to view the data, identify faulty scans, remove glitches/Bragg reflections from the spectra and to average multiple XAS spectra into one. The averaged XAS spectra were then opened by PySpline^[4]^, to normalize, remove the spline, and obtain the weighted EXAFS spectrum and its Fourier-transform. This data set was imported by Microsoft Excel 365, to adjust the k-weighting of the respective column. The final data was then fitted in the software EXAFSPAK^[5]^ using path files generated by FEFF10^[6]^ based on reported crystal structures. For all fits, the Marquardt curve fitting algorithm was used. In some cases, the fits were verified by fitting the same spectrum with the same parameters in Artemis.

Any XAS/EXAFS spectra shown in the text section were created using Microsoft Excel 365 or Origin Pro 2021b, spectra in the appendices were generally using the default export settings of the respective software (EXAFSPAK or Athena).

## **^64^Copper uptake measurements**

To evaluate the uptake of ^64^Cu^2+^ with and without patellamides, three different target organisms were chosen, *Prochloron didemni* (*P.d.*) collected during the field trips, *Nannochloropsis occulata* (*N.o.* due to its availability and previous toxicity measurements done on *Nannochloropsis spp.*) and *Anabaena sp* (for this species the measurements did not work, as they could not be properly drained by centrifugation). The measurements were performed within 3 days of the return from the sample collection trip to ensure *Prochloron* was as vital as possible, which was confirmed by evaluating the photosynthesis activity and yield using a Walz Mini-PAM. For all three organisms a suitable cultivation medium was chosen (reef water for *Pd,* BG11 medium for *Anabaena* and *N.o.*), to the medium for half the samples a filtered and denatured extract from the ascidian cloaca, rich in different patellamides was added. The ^64^CuCl_2_ solution (ultrapure water, no carrier added) was produced on an 18 MeV Twin cyclone cyclotron (IBA, Belgium) at the Australian Institute for Bioengineering and Nanotechnology, University of Queensland (St Lucia, Queensland) through the ^64^Ni(p,n)^64^Cu nuclear reaction.

Each species was split up into four separate Eppendorf vials, centrifuged down and the supernatant removed. 10 µL of a copper solution containing ^64^Cu^2+^ diluted to 100-200 MBq/mL as well as 1mM CuSO_4_ (to ensure a sufficient concentration of Cu^2+^ for the formation of the dicopper(II) complex) was added to 1mL of culture medium for each sample. For each species, for two of the samples the patellamide-rich medium was used, for two the medium without patellamides (due to limited availability of the naturally sourced and not cultivatable *P. didemni* and cloacal extract*,* the measurements were only performed in duplicate instead of triplicate, a repetition would have been considered if there was a significant big deviation between the replicates). After incubation at 30°C (30°C instead of 37°C was chosen as incubation temperature, to provide more natural conditions for the sensitive *Prochloron* cultures) for 2h the samples were centrifuged, and the uptake of ^64^Cu^2+^ was evaluated by measuring the count rates of the supernatant and pellet measured separately using a Wizard 2480 Automatic Gamma counter**.** The samples were washed by resuspension in medium, followed by centrifugation and removal of the supernatant. Then to each sample the respective medium with and without patellamides was added once more, this time without added ^64^Cu^2+^. After another incubation at 30°C for 2h the samples were centrifuged again and the supernatant and pellet measured separately again to evaluate how much copper-64 was released by the specimen.

**Determination of intracellular pH**

The freshly harvested cyanobacteria were directly incubated with BCECF/AM, initially the ideal incubation conditions were tested by first incubating for 30 minutes at room temperature and by subjecting the cyanobacteria to an acidic shock (1 minute in 1M HCl), followed by 30 minutes incubation with BCECF/AM – and comparing different concentrations of BCECF/AM (25 µM/L, 10 µM/L, 5 µM/L). As the cyanobacteria did not survive the acid treatment, future cultures were directly incubated with BCECF/AM. While all concentrations gave a measurable signal, 10 µM/L was considered the ideal concentration.

All plate reader measurements were performed at the IMB of the University of Queensland, Brisbane, in 8-fold repetition on a 96-well plate. The measurements were performed using a Tecan Spark Multimode plate reader. All confocal microscopies were performed using the Zeiss Axiovert 200 Inverted Microscope Stand equipped with a LSM 710 Meta Confocal Scanner in cooperation with Dr. Kathy Wu in the IMB microscopy facility.

For the measurement of the intracellular pH value in *P.d.*, the ratiometric fluorescent dye BCECF/AM (2ʹ,7ʹ-bis-(carboxyethyl)-5(6ʹ)-carboxyfluoreszein-acetoxymethylester), was used. While the BCECF acid is the luminous signaling form of the dye, the acetoxymethyl ester is membrane permeable and hydrolyzed by esterases inside the cells, allowing easy loading of the dye into the cells as well as a selective luminosity inside the cell. Two living specimens of *L.p.* were transported from Heron Island to the laboratory in Brisbane, to allow for the collection of fresh and intact specimens of *P.d.*, since the cyanobacteria often die within 1-3 days when separated from their host. Their vitality was confirmed experimentally by following their photosynthetic efficiency, using a PAM (Pulse Amplitude Modulation) chlorophyll fluorometer. The cyanobacteria were freshly collected from the animal and incubated with BCECF/AM as described by the manufacturer^[7]^, once at room temperature for 30 minutes, or after subjecting the cyanobacteria to an acid shock (50mM HCl for 5 minutes). While the acid shock led to the death of the cyanobacteria, making these measurements unusable, the incubation at room temperature with BCECF/AM gave a sufficient load of the dye into the cells and left the organisms alive. As reference for the measurements, BCECF/AM was dissolved in a lysate of *E. coli,* which was set to different pH values, to create a set of reference spectra and the respective regression curve for the dataset (see Figure S9). The experimental procedure is described in more detail in the Experimental Section.

To verify the success of the loading procedure and to confirm, that the signal measured originates from within the cyanobacteria and not from the surrounding aqueous solvent, the incubated cells were measured with a confocal fluorescence microscope (Figure 10). As evident from Figure S10, using the same excitation and emission settings, the negative control only shows a weak, barely noticeable emission, which can be attributed to a slight autofluorescence. The positive control shows a clear and highly intense fluorescence of BCECF within the targeted cells.

The loading was compared for a 1:40, 1:100, and 1:200 dilution of the 1 mM/L BCECF/AM stock solution, and the spectra show very similar features and ratios at all three dilutions. For further assays and for the microscopy the 10 µM concentration was chosen, as the 1:200 dilution showed a worse signal-to-noise ratio than the higher concentrations with less pronounced excitation maxima, while the 1:40 dilution gave a very similar signal to the 1:100 dilution and thus the lower concentration was preferred. The ratios for the emission at 560 nm with the excitation at 504 nm to the excitation at 440 nm for all three dilutions were around 2.1 and the ratio for the emission at 560 nm with the excitation at 504 nm to the excitation at 460 nm was around 1.32 (Table S2). The ratios were determined for both excitation wavelengths, as the instrumentally preferred wavelength uses 440 nm for technical reasons, while the measured spectra showed the second maximum at 460 nm.

**Table S2.** Overview of the excitations ratios of the spectra shown in Figure S8.

| Dilution | Concentration | Ratio  F(504)/F(440nm) | Ratio  F(504)/F(460nm) |
| --- | --- | --- | --- |
| 1:40 | 25 µM | 1.870531 | 1.194236 |
| 1:100 | 10 µM | 2.142207 | 1.380411 |
| 1:200 | 5 µM | 2.053777 | 1.379613 |
|  | **Average** | **2.097992** | **1.318087** |

The pH-dependence of the fluorescence excitation ratios shows a nearly linear trend (Appendix A-pH 2). However, the accurate determination of the pH value was performed based on Equation 2 and 3, which describe the relationship between proton concentration and its logarithm and fluorescence ratios, with **R** being the ratio between the fluorescence emission at 560nm upon excitation at λ_1_ = 504nm to the emission upon excitation at λ_2_ = 460nm in the samples; **R_A_** and **R_B_** are the respective ratios at the acidic (R_A_) and basic (R_B_) end of the reference titration and F_A(λ2)_/ F_B(λ2)_ are the absolute values of the fluorescence at 560nm upon excitation at λ_2_.

$$\left[ H^{+} \right]=K_{a}\frac{\left( R-R_{A} \right)}{\left( R_{B}-R \right)}\times\frac{F_{A\left( \lambda_{2} \right)}}{F_{B\left( \lambda_{2} \right)}} (1)$$

**Equation 1.** Relationship between H-concentration and the fluorescence ratios

$$pH={pK}_{a}-\log\left( \frac{\left( R-R_{A} \right)}{\left( R_{B}-R \right)}\times\frac{F_{A\left( \lambda_{2} \right)}}{F_{B\left( \lambda_{2} \right)}} \right) (2)$$

**Equation 2.** Relationship between the pH and the fluorescence Ratios

The resulting value for the pH, as determined by this method, is **6.9 (±0.1).** Interestingly, this value is slightly lower than in most other cyanobacteria and lower than the intracellular pH for carbon-fixating cyanobacteria discussed by Mangan et al.^[8]^. The intracellular pH in cyanobacteria can vary depending on the surrounding light irradiation^[8]^, but as the cyanobacteria for this experiment were not dark-adapted, the pH should have been at the maximum level. This might indicate that *Prochloron* has an alternative way to ensure sufficient availability of carbon for its high levels of photosynthesis^[9]^.

**Bioactivity of patellamides and copper**

The bioactivity of patellamides has been evaluated with several human cell lines, and its toxicity towards potential predators and infectious microorganisms was evaluated. In all published experiments, only the metal-free patellamide was used, and apart from some interactions with membrane proteins in human leukemia cell lines^[10]^, little activity was found. A possible positive or negative effect on the vitality of photosynthetic organisms such as algae or cyanobacteria has not yet been tested. To check these properties, a series of experiments was devised, where reference algae (*Nannochloropsis gaditana* or *Nannochloropsis salina*) were subjected to different concentrations of copper(II), to metal-free patellamides and to a combination of copper(II) and patellamides. The vitality of the algae was evaluated using a PAM chlorophyll fluorometer, as the fluorescence parameters of algae are known to be a good indicator of their vitality ^[11]^.

Different parameters can be considered when testing the vitality of the algae. Firstly, the maximum photosynthesis rate **F_m_** can be measured by saturating the chlorophyll excitation, closing all reaction centers of a dark-adapted sample (kept in complete darkness for at least 1 hour) sample. This parameter indicates the maximum level of excitable chlorophyll i.e., its content or the number of algae. Secondly, the current or variable chlorophyll fluorescence **F_V_** can be observed (indication of the photosynthetic rate at a given moment). As third option, the minimum fluorescence **F_0_** can be measured during the minima of the excitation pulses. This parameter gives an indication of the low-light or rest fluorescence in the absence of an excitation pulse with all reaction centers open and unexcited. Lastly, the photosynthesis yield **Y** = (F_V_ (F_m_ – F_0_)/ F_m_) can be determined, which indicates the efficiency of the photochemical reactions in photosynthesis II.

To algae of the species *Nannochloropsis salina* patellamides either only patellamide (25 µM/L or 50 µM/L) or copper(II) (1ppm or 2ppm) or both (25 µM/L or 50 µM/L asc + 1ppm copper) were added. Interestingly, when plotting the relative change of the maximum fluorescence (F_m_ of treatment compared to blank, see Figure S4), a significant increase in maximum fluorescence F_m_ can be observed, already within few hours after treatment. This may be caused by an increase in the number of algae or the effective number of reactive centers in PS2 within the culture. As evident, there is hardly any difference between the samples with and without copper(II) added, which is most likely because copper(II) at these low concentrations is often already contained in the growth medium of marine algae.


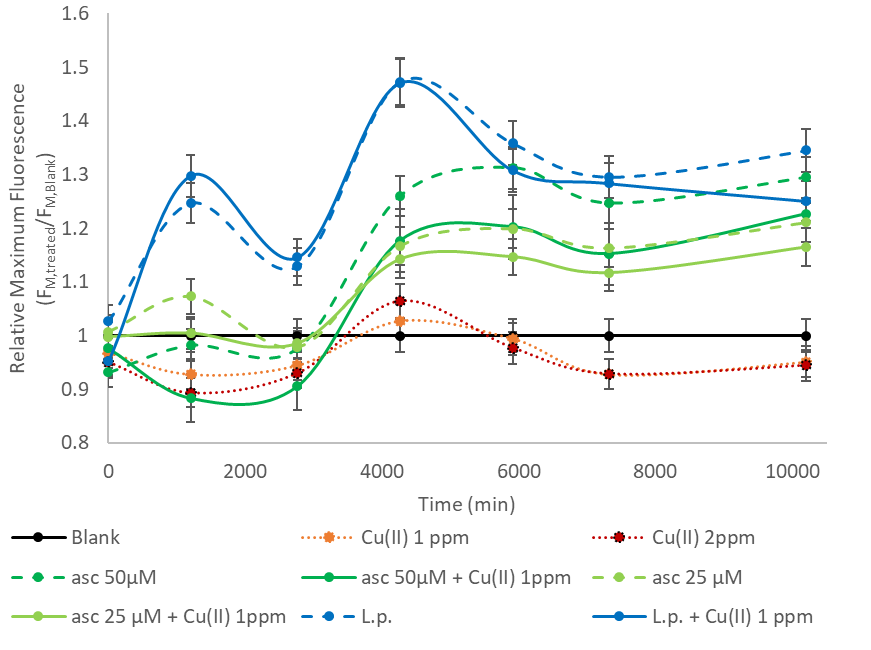


**Figure S4.** Change of the relative maximum chlorophyll fluorescence of the dark-adapted samples, shown relative to the values for the blanks. All measurements are averaged over 3 samples, the average standard deviation over all samples is ~3%. The two Asc series correspond to concentrations of 25 µM/L (Asc2, bright green) and 50 µM/L (dark green). In the samples labeled Pd, an extract from filtered cloacal water, collected from *Lissoclinum patella*, was directly added to the samples.

At these low concentrations of copper(II), no change between the treated cultures with and without copper(II) was noticeable. However, with algae of the same species at higher concentrations of copper(II) (50 - 100ppm), this was different (Figure S5). Algae of the same genus *Nannochloropsis* (this time the species *Nannochloropsis gaditana*) were treated with either copper (50 or 100 ppm), ascidiacyclamide, or PAOO_SS_ (53 µM), or both. At concentrations of 100 ppm, the algae died within 2 days, while at 50 ppm, they showed a decrease in fluorescence rate but recovered. While the metal-free patellamides did not show any negative effect, together with copper(II), the algae died as quickly as with the higher, lethal concentration of copper(II). The decrease in vitality is evident from the drop in variable chlorophyll fluorescence F_V_ measured using a PAM. In addition, the effect could usually be observed by the naked eye, as the deceasing samples changed their color from green to colorless-brown.

While the negative effects observed at this significantly higher concentration of copper(II) are most likely of little relevance for the biological system, they contradict one older hypothesis ^[12]^ on a possible biological function of the patellamides, to protect the cyanobacteria from the toxic effects of copper.

The experiments with the two different concentration ranges of copper(II) have shown, that at the physiological concentration of copper(II), patellamides do not decrease and eventually enhance the efficiency of the light reaction of PSII of an algae culture. In contrast, at excess concentrations of copper(II), a detrimental effect can be observed, enhanced by the patellamides. A possible explanation for this is that the patellamides as small cyclic peptides with short hydrophobic side chains pass the membranes in sufficient amounts. After binding copper(II), they can shield the charge of copper(II) and possibly enhance the transport of copper into the algae, explaining the detrimental effects observed in the first series of experiments. At lower copper(II) concentrations, the patellamides might be used to transport limited available carbonate through the membranes, thus increasing the carbon fixation activity of the algae as part of the photosynthesis cycle. However, it can not be excluded that the different results are in parts caused by the two different subspecies of the genus *Nannochloropsis* but due to the high similarity between the two cultures only a minor effect would be expected.

**Figure S5.** Comparison of the relative variable fluorescence (F_V_/F_V,Blank_) of the samples under illumination (a lamp simulating light composition in the sea), relative to the control culture. All curves represent the average of 2-3 samples. Both asc (ascidiacyclamide) and PANOSS (patellamide analogue with oxazole heterocycles and S,S side chain stereochemistry) were added to concentrations of 53 µM/L. The same concentration of copper the algae recovered from became lethal when added with the patellamides.

**Parallel artificial membrane permeability assays (PAMPA)**

PAMPA pre-coated assay plates were obtained from Corning® and were the BioCoat® Pre-coated PAMPA Plate System. The initial test compound was dissolved in dimethyl sulfoxide (DMSO) to produce a 5 mM stock solution and then diluted in phosphate-buffered saline (PBS) to give a 1% DMSO or 50 µM final solution.

PBS (300 µL) was added to the basolateral side, the membrane chamber was set. Test compound (200 µL) was applied to the apical side the membrane and incubated at 37°C in an atmosphere of 95% humidity and 5% CO2 for 4 hours. After the incubation, samples from the apical (150 µL) and basolateral sides (250 µL) were collected.

The concentration of the test compounds in samples from the apical and basolateral sides were quantified using UPLC/MS (Shimadzu) with a Phenomenex 5 µm C18 / 300 Å / 150 x 2 mm LC column. Concentration was determined by area under the curve with a five-point linear standard curve (0.2, 0.6, 1.9, 5.6, and 16.7 µM). The % recovery was determined from peptide concentrations recovered from apical and basolateral samples, relative to the starting concentration. The apparent permeability coefficient (Papp) of the test compounds were calculated using the equation from the Caco-2 cell permeability assay.

**Caco-2 cell membrane permeability assay**

Caco-2 cell membrane permeability assays were performed using previously described methods.^[13]^ The human colon carcinoma Caco-2 cell line was obtained from American Type Culture Collection (ATCC, USA). Cells were cultivated and maintained in T-175 flasks in Dulbecco’s Modified Eagle Medium (DMEM) supplemented with 10% (v/v) Fetal Bovine Serum (FBS), 1% (v/v) non-essential amino acids, and Penicillin/Streptomycin at 37° C in an atmosphere of 95% humidity and 5% CO2. Cells were subcultured when they reached 80% confluence.

Cells were seeded at a concentration of 4 × 105 cells/mL (0.75 mL, 3 × 105 cells/well) on Transwell 12-well plates (Corning) in DMEM supplemented with 10% (v/v) FBS, 1% (v/v) non-essential amino acids, 0.4 µg / mL puromycin and cultivated for 8-10 days. Transepithelial/endothelial electrical resistance (TEER) was measured to confirm the integrity of the Caco-2 cell monolayer for the assay (> 400 Ω).

The 50 µM test compound in 10 µM-Lucifer yellow in Hanks’ balanced salt solution (HBSS) was prepared as an initial solution. Test compounds were applied to the apical side of the Caco-2 cell monolayers and incubated at 37°C in an atmosphere of 95% humidity and 5% CO2 and subjected to 55 rpm orbital shaking. After 90 minutes, samples from the apical and basolateral sides were collected.

The concentrations of the test compounds in samples from the apical and basolateral sides were quantified using UPLC/MS (Shimadzu, Phenomenex 5 µm C18 / 300 Å / 150 x 2 mm LC column). The recovery of each compound was calculated for the apical and basolateral sides. The apparent permeability coefficient (Papp) of the test compound and its recovery were calculated.

$$Recovery rate: R \left( \% \right)=\frac{{(C}_{A}\times V_{A}+C_{B}\times V_{B})}{C_{0}\times V_{A}}\times100$$

where CA and CB are the apical and basolateral concentrations (µM), VA and VB are the apical and basolateral volumes (mL), and C0 is the initial concentration (µM).

The apparent permeability coefficient (Papp) was calculated using the following equations and where A is the membrane surface area (cm2):

$$Papp (cm/s) = \frac{-ln(1-\frac{C_{B}}{C_{e}})}{A \times\left( \frac{1}{V_{A}}+\frac{1}{V_{B}} \right)\times time(s)}$$

$$C_{e}= \frac{(C_{A}\times V_{A}+C_{B}\times V_{B})}{V_{A}+V_{B}}$$

**Statistics (error bars in the various Figures)**

Figure 1: standard deviations from 3 replicates

Figure 3: standard deviations from 2 replicates

Figure 4: Caco-2: standard deviations from 3 replicates

Figure S4: standard deviations from 3 or more replicates

Figure S6: standard deviations from 3 replicates

Figure S7: standard deviations from 3 replicates

# **Supplementary Data**

**qPCR**

**Figure S6.** qPCR relative expression rates of the patellamide genes patA-patG relative to housekeeping gene 16S after injection of different metals and concentrations of copper (0/5/10/20 mg/mL).The naming in the X-axis consits of specimen ID (Cu: ascidian only injected with Cu, Fe: ascidian injected with Cu, Fe, Zn, V: Injected with Cu, V, Zn), Injected area number (1-4) and copper concentration or element injected (0/5/10/20/Z/V) It is noteworthy that for some animals and genes zinc (R Fe_4-Z / R V_4 Z) has led to an increased signal as well. However, this is not reflected in the measured final concentrations.

**Table S3. ^64^Cu experiments: raw data.**

**Figure S7** (subset of Figure S6 with data for Cu^II^ only)**.** qPCR relative expression rates of the patellamide genes patA-patG relative to housekeeping gene 16S after injection of different concentrations of copper (0/5/10/20 mg/mL). All genes show significantly increased expression rates compared to the blank, except for patE, the gene encoding the precursor amino acids which are turned into patellamides by the enzymes encoded in the other genes. The samples were taken 24 hours after injection, the precursor might have increased strongly at first, and upon reaching target concentration been downregulated again.

| **Uptake** |  |  |  |  |
| --- | --- | --- | --- | --- |
| **Pellets** |  |  |  |  |
| **SampleID** | Time | Counts | CPM | Error |
| Prochloron didemni+1 | 2:27:59 PM | 2218495 | 2422440 | 0.06% |
| Prochloron didemni+2 | 2:29:13 PM | 2198804 | 2399082 | 0.06% |
| Prochloron didemni01 | 2:30:27 PM | 2542633 | 2810601 | 0.06% |
| Prochloron didemni02 | 2:31:41 PM | 2448753 | 2697316 | 0.06% |
| Nanochloropsis occulata with patellamide 1 | 2:32:55 PM | 52015 | 52077 | 0.44% |
| Nanochloropsis occulata with patellamide 2 | 2:36:38 PM | 64322 | 64453 | 0.39% |
| Nanochloropsis occulata without patellamide 2 | 2:37:52 PM | 16054 | 16054 | 0.79% |
| Nanochloropsis occulata without patellamide 2 | 2:39:06 PM | 11807 | 11805 | 0.92% |
|  |  |  |  |  |
| **Supernatant** | |  |  |  |
| **SampleID** | Time | Counts | CPM | Error |
| Prochloron didemni+1 | 14:59:52 | 646945 | 663063 | 0.12% |
| Prochloron didemni+2 | 15:01:05 | 698842 | 718798 | 0.12% |
| Prochloron didemni01 | 15:02:19 | 272635 | 275500 | 0.19% |
| Prochloron didemni02 | 15:03:33 | 284703 | 287833 | 0.19% |
| Nanochloropsis occulata with patellamide 1 | 15:04:47 | 2689049 | 2987861 | 0.06% |
| Nanochloropsis occulata with patellamide 2 | 15:06:00 | 2623401 | 2908582 | 0.06% |
| Nanochloropsis occulata without patellamide 2 | 15:07:15 | 2742802 | 3054593 | 0.06% |
| Nanochloropsis occulata without patellamide 2 | 15:08:29 | 2746807 | 3059690 | 0.06% |
|  |  |  |  |  |
|  |  |  |  |  |
| **Release** |  |  |  |  |
| **Pellets** |  |  |  |  |
| **SampleID** | Time | Counts | CPM | Error |
| Prochloron didemni +1 | 4:52:38 PM | 1905509 | 2055816 | 0.07% |
| Prochloron didemni +2 | 4:53:53 PM | 1938329 | 2093817 | 0.07% |
| Prochloron didemni 01 | 4:55:06 PM | 2194858 | 2394433 | 0.06% |
| Prochloron didemni 02 | 4:56:20 PM | 2231954 | 2437610 | 0.06% |
| Nanochloropsis occulata with patellamide 1 | 4:57:35 PM | 12490 | 12488 | 0.89% |
| Nanochloropsis occulata with patellamide 2 | 4:59:24 PM | 19346 | 19348 | 0.72% |
| Nanochloropsis occulata without patellamide 2 | 5:00:39 PM | 10440 | 10435 | 0.98% |
| Nanochloropsis occulata without patellamide 2 | 5:01:53 PM | 7167 | 7165 | 1.18% |
|  |  |  |  |  |
| **Supernatant** | |  |  |  |
| **SampleID** | Time | Counts | CPM | Error |
| Prochloron didemni +1 | 17:09:01 | 62274 | 62394 | 0.40% |
| Prochloron didemni +2 | 17:10:16 | 60628 | 60743 | 0.41% |
| Prochloron didemni 01 | 17:11:29 | 55643 | 55753 | 0.42% |
| Prochloron didemni 02 | 17:12:43 | 50659 | 50731 | 0.44% |
| Nanochloropsis occulata with patellamide 1 | 17:13:57 | 32786 | 32808 | 0.55% |
| Nanochloropsis occulata with patellamide 2 | 17:15:11 | 29109 | 39138 | 0.51% |
| Nanochloropsis occulata without patellamide 2 | 17:16:25 | 3249 | 3246 | 1.75% |
| Nanochloropsis occulata without patellamide 2 | 17:17:38 | 3600 | 3598 | 1.67% |

**Intracellular pH value determination**

**Figure S8**. Excitation spectrum, measuring the emission intensity at 560 nm of Prochloron didemni incubated with three different concentrations of BCECF at variable excitation wavelengths. The shown spectra are averaged over 8 samples.

**Figure S9.** Spectra for the reference measurements of the pH-dependence of the emission intensity at 560nm of 0.05mM BCECF/AM in lysated cells at variable excitation.


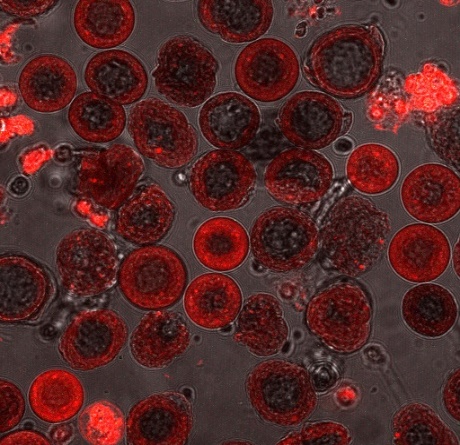

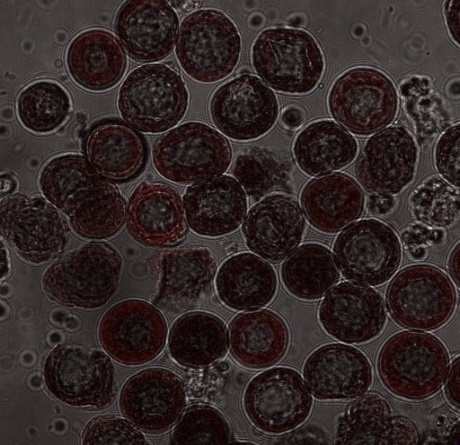

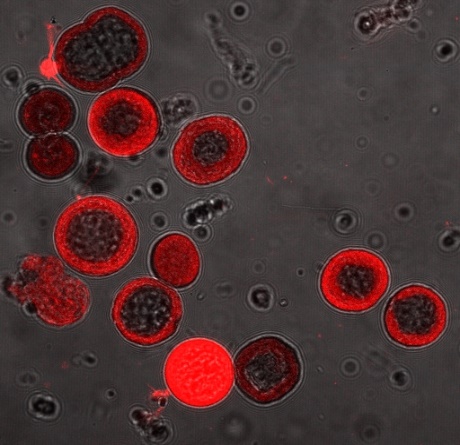


**Figure S10.** Confocal fluorescence images of *Prochloron didemni*. left: negative control (unloaded cells) middle, right: Cells loaded with 0.01mM of the fluorescent radiometric dye BCECF/AM.


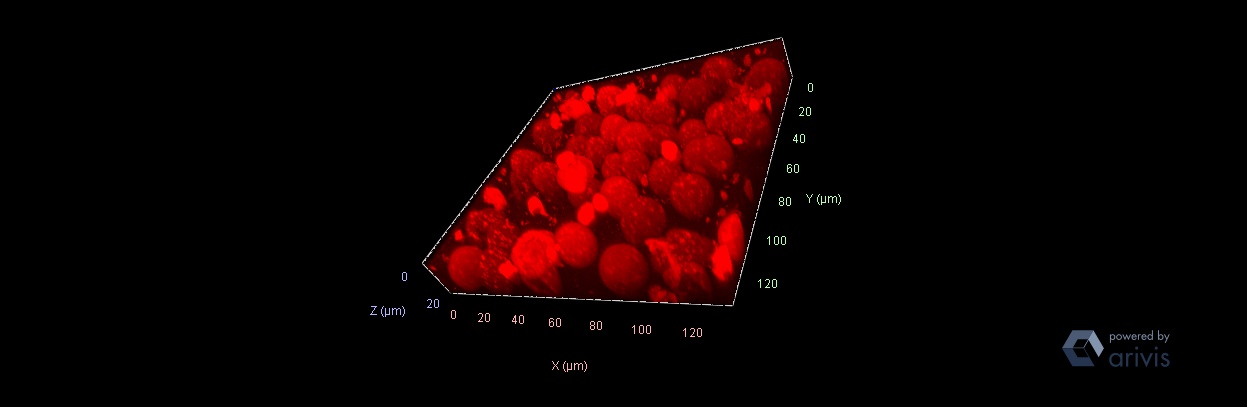


**Figure S11**. 3D confocal fluorescence microscopy scan of *Prochloron didemni*, loaded with BCECF-dye. The intensity of the red color corresponds to the total emission between 500 and 630nm upon excitation at 488nm.

**Table S4:** Raw data of the BCA assay and the PHERAstar measurement for each of the ascidian extracts.

|  | BCA ASSAY | Referenced blank | | PHERAstar |  |  |  |  |  |
| --- | --- | --- | --- | --- | --- | --- | --- | --- | --- |
| Sample ID |  | BCA |  |  | PHERA |  |  |  |  |
| Cu_1-0 | 2.469 | 0.44790336 |  | 9.967 | 0.59677274 |  |  |  |  |
| Cu_2-5 | 5.221 | 0.94704348 |  | 18 | 1.07774751 |  |  |  |  |
| Cu-3-10 | 3.803 | 0.68990242 |  | 11.425 | 0.68407029 |  |  |  |  |
| Cu_4-20 | 4.537 | 0.82308144 |  | 15.159 | 0.90764303 |  |  |  |  |
| Fe_2-Cu | 3.872 | 0.70241791 |  | 11.899 | 0.71245098 |  |  |  |  |
| Fe_3-F | 2.021 | 0.36662261 |  | 8.63 | 0.51672006 |  |  |  |  |
| Fe_4-Z | 3.718 | 0.67453418 |  | 12.461 | 0.74610065 |  |  |  |  |
| V_1-0 | 2.252 | 0.40846195 |  | 9.055 | 0.54216687 |  |  |  |  |
| V_2-Cu | 5.804 | 1.05295652 |  | 15.403 | 0.92225249 |  |  |  |  |
| V_3-V | 3.298 | 0.59828461 |  | 11.973 | 0.71688172 |  |  |  |  |
| V_4-Z | 2.474 | 0.44882433 |  | 9.459 | 0.56635632 |  |  |  |  |
|  |  |  |  |  |  |  |  |  |  |
| **Averaged Data** |  |  |  |  |  |  |  |  |  |
|  | BCA ASSAY | Referenced blank | |  |  | PHERAstar |  |  |  |
| Sample ID |  | AVERAGED | Relative |  |  |  | PHERA |  |  |
| Blank | 2.46909339 | 2.36038186 | 1 | 0 |  | 9.967 | 9.511 | 1 | 0 |
| Cu | 5.22063241 | 4.64752961 | 1.96897362 | 0.96897362 |  | 18 | 14.3772 | 1.51163915 | 0.51163915 |
| Fe | 3.80312733 | 2.0210285 | 0.85622947 | -0.1437705 |  | 11.425 | 8.63 | 0.90737041 | -0.0926296 |
| V | 4.53728443 | 3.29807881 | 1.39726494 | 0.39726494 |  | 15.159 | 11.973 | 1.25885816 | 0.25885816 |
| Zn | 3.8721197 | 3.09628964 | 1.31177488 | 0.31177488 |  | 11.899 | 10.96 | 1.15234991 | 0.15234991 |

**HPLC-MS-data (injection of metal ion solutions into the ascidians)**

Due to the large file size of the dataset, all graphs here were exported as image from Sciex Multiquant.

**Figure S12.** Overlay of the retention time region in the HPLC-MS corresponding to the patellamides, of the specimen injected with water (black), copper (red), zinc (green) or iron/vanadium (blue/bright blue). As evident the injection of copper led to the most pronounced increase in patellamide concentration.

**Figure S13.** Overlay of the retention time corresponding to patellamide A in the HPLC-MS of specimen A injected with copper. All concentrations of copper have led to an increased amount of patellamide A, however the injection of 5 and 10 mM solution (red/pink), showed a stronger increase than the injection of the 20mM solution.

**Figure S14.** Overlay of the retention time corresponding to patellamide A in the HPLC-MS of specimen B injected with water (blue), copper(pink), vanadium (red) or zinc (green). Only copper led to a significant increase in patellamide A concentration.

**Figure S15.** Overlay of the retention time corresponding to patellamide A in the HPLC-MS of specimen C injected with water (blue), copper(pink), iron (red) or zinc (green). Only copper led to a significant increase in patellamide A concentration, zinc slightly increased the concentration as well.

**Figure S16.** Overlay of the retention time corresponding to ascidiacyclamide in the HPLC-MS of specimen A injected with different concentrations of copper. Only 5mM and 10mM solutions of copper led to an increase in ascidiacyclamide concentration.

**Figure S17**. Overlay of the retention time corresponding to ascidiacyclamide in the HPLC-MS of specimen B injected with water (blue), copper(pink), vanadium (red) or zinc (green). Only copper led to a significant increase in ascidiacyclamide concentration.

**Figure S18.** Overlay of the retention time corresponding to ascidiacyclamide in the HPLC-MS of specimen C injected with water (blue), copper(pink), iron (red) or zinc (green). Only copper led to a significant increase in ascidiacyclamide concentration, zinc showed an increase as well.

**Figure S19**. Overlay plots of the retention time corresponding to patellamide C/F in the HPLC-MS for all three specimen, A (left), B (right), C (bottom) – showing the same relative differences as found for ascidiacyclamide (Figures S16-S18).

## **XAS Data**

**
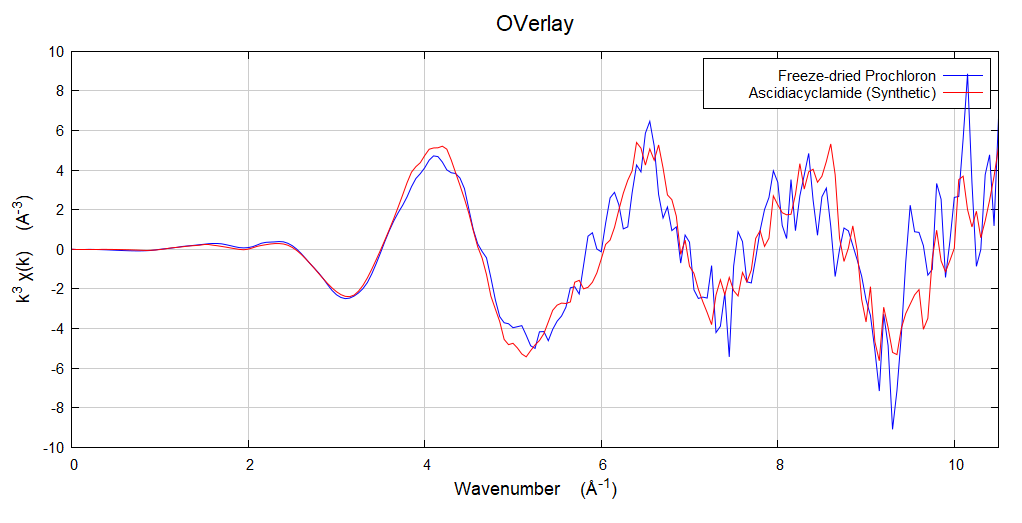
**

**Figure S21.** Larger version of the overlay (shown in Figure 1 of the manuscript) of the k-weighted EXAFS of the measured biological sample (black) and the fitted EXAFS (red) in the (*k* range of k = 2-11.5). Measured at the SuperXAS beamline of the PSI in Switzerland using an SDD. The biological sample data was merged over 20 scans of 20 minutes at different spots.

**Figure S20**. Larger version of the overlay (shown in Figure 1 of the manuscript) of the EXAFS-Signal from freeze-dried *Prochloron didemni* (collected during the field experiments in 2022), with the EXAFS of synthetically produced Cu^II^_2_-ascidiacyclamide (in DMSO:H_2_O, 4 mM/L, with Bu_4_NxMeOH with a ratio Asc/Cu/base of 1/2/5) complexes. Measured at the SuperXAS beamline of the PSI in Switzerland using an SDD. The biological sample data was merged over 20 scans of 20 minutes at different spots, the synthetic sample data was merged over 6 scans of 20 minutes at different spots.

**Figure S22.** Overlay of the EXAFS-spectra of freeze-dried prochloron (blue) and synthetic ascidiacyclamide (red) with a *k*-weighting of 3.


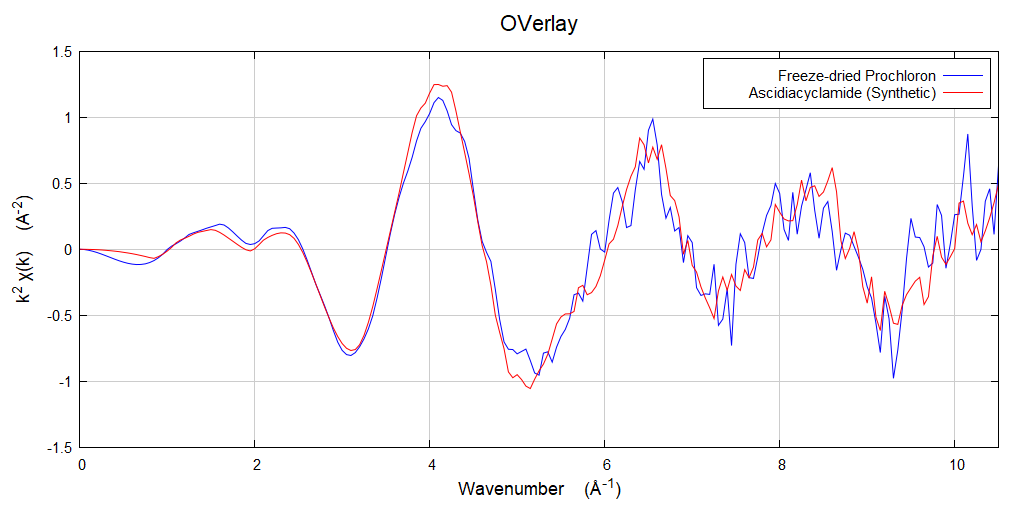


**Figure S23**. Overlay of the EXAFS-Spectra of freeze-dried prochloron (blue) and synthetic ascidiacyclamide (red) with a *k*-weighting of 2 (created in Athena).


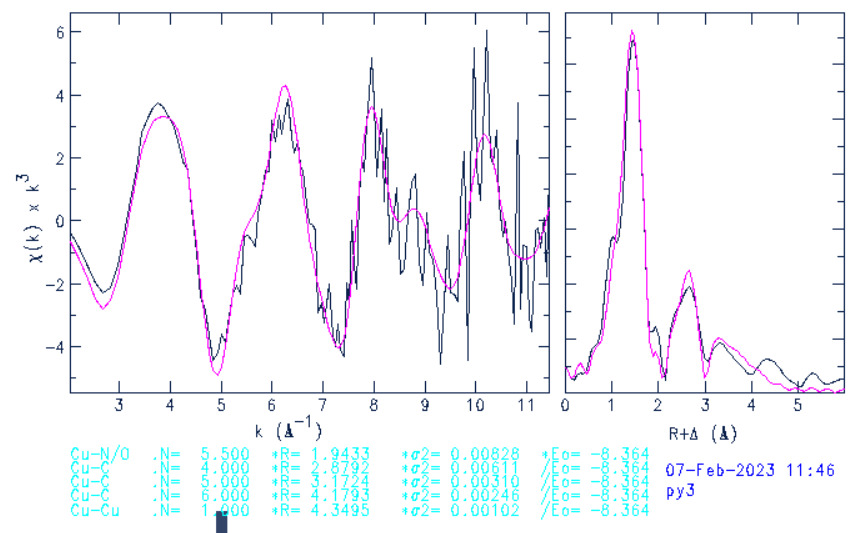

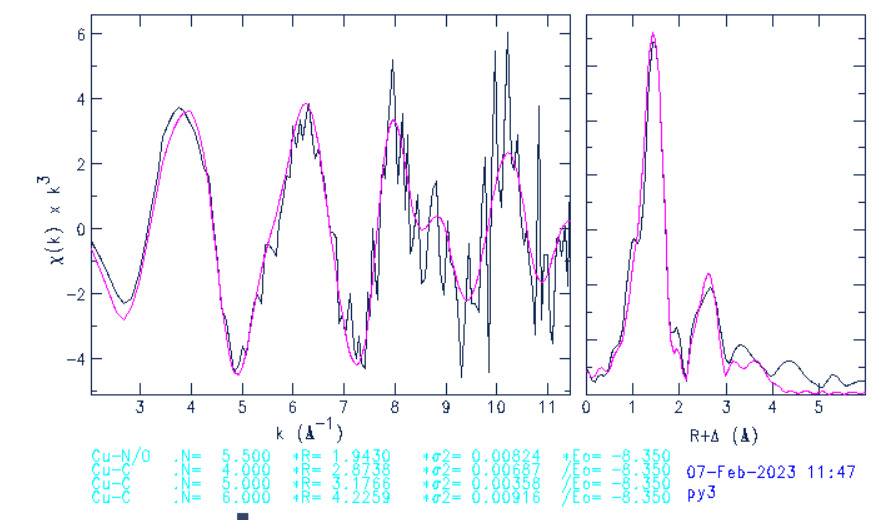


**Figure S24.** The fits of the EXAFS spectrum of the biological sample with (top) and without (bottom) inclusion of the copper-copper path. While the noise in the high-k (>7) region makes a clear interpretation of long paths difficult, it is evident, that without the copper-copper path, the fit lacks intensity between 3-4 Å. It must be noted that the distances in the Fourier-transformed spectra on the right are not drawn phase-corrected and thus the distances on the X-axis correspond to longer real distances (figures created in EXAFSPAK).


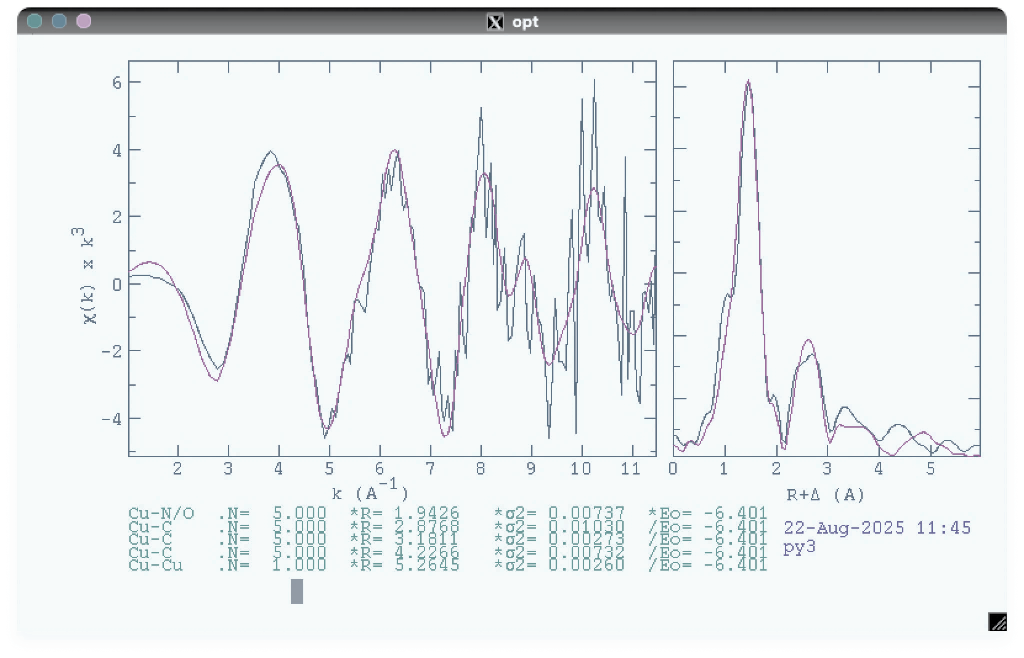

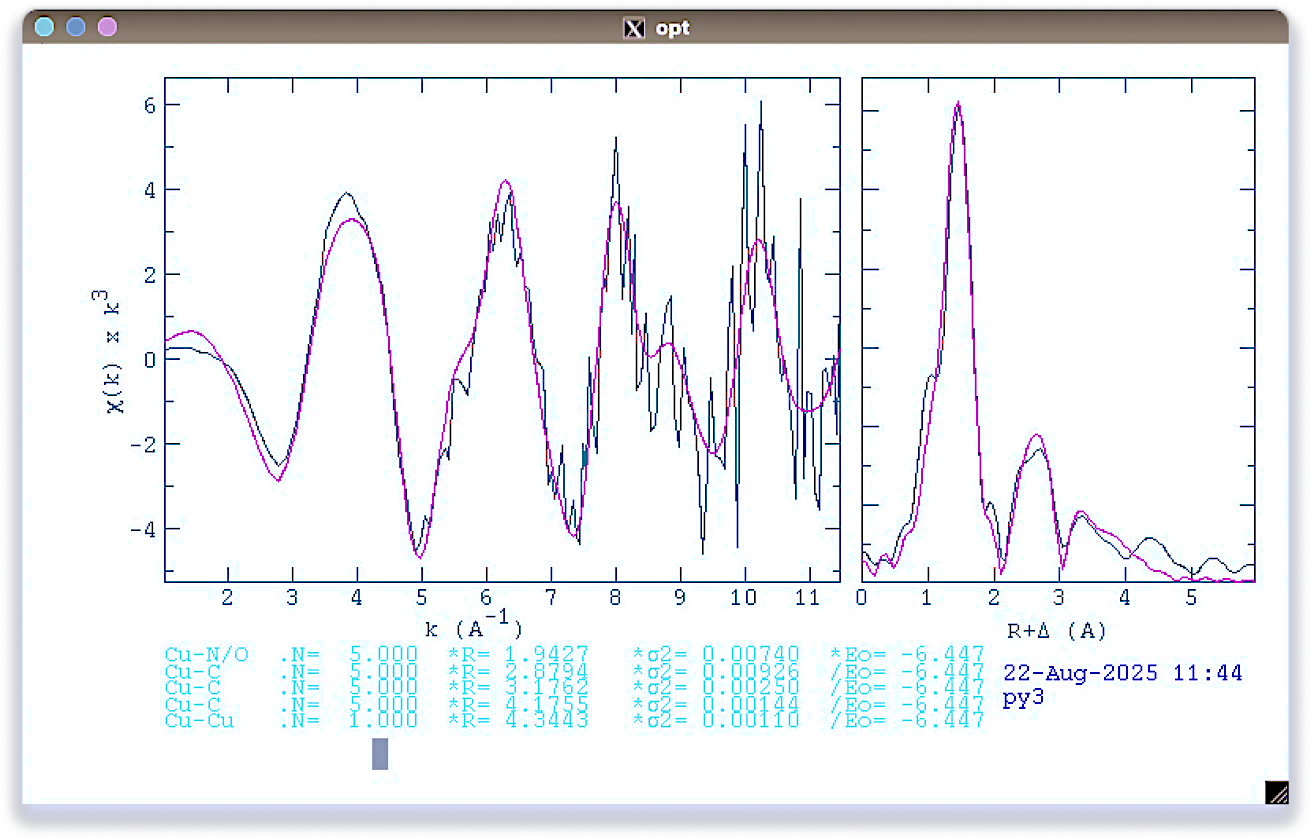

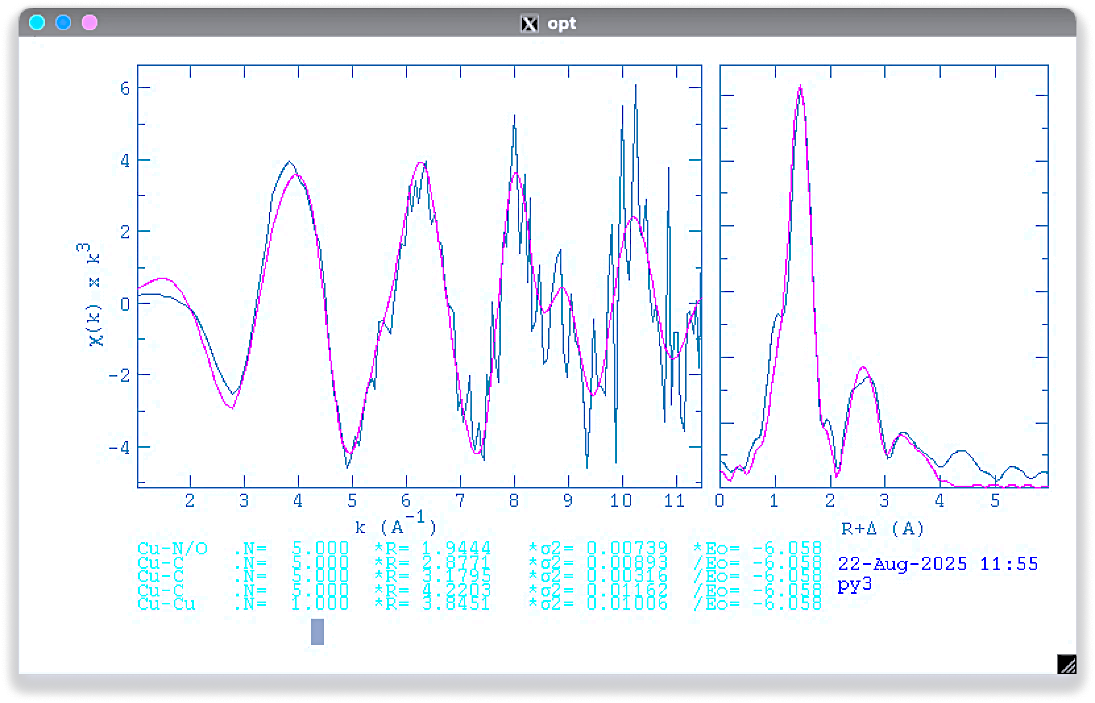


**Figure S25.** The fits of the EXAFS-spectrum of the biological sample with the same fitting parameters of C/N/O except varying Cu-Cu distances of 3.85Å (top, as experimentally suggested by EPR), 4.34Å (middle, suggested by the crystal structure) and 5.26Å (bottom, the DFT-calculated distance for an unbridged complex). In the highlighted region of the graphs, the 3.8Å fit shows the best fit to the experimental data, followed by the quite similar 4.3Å fit, while the 5.2Å fit shows larger deviations, especially in the (phase corrected) Fourier transform above 3Å.

The following images were created using TOPPview of the mass spectra files, measured at the Sciex TripleTOF 5600 and 6600. Each image consists of the chromatogram at the top (y axis: counts, x axis: time [min]), and the mass distribution on the right (y: m/z, x: relative quantity), leading to the 2D plot at the bottom left (x: time [min], y: m/z, the color shows the relative quantity).


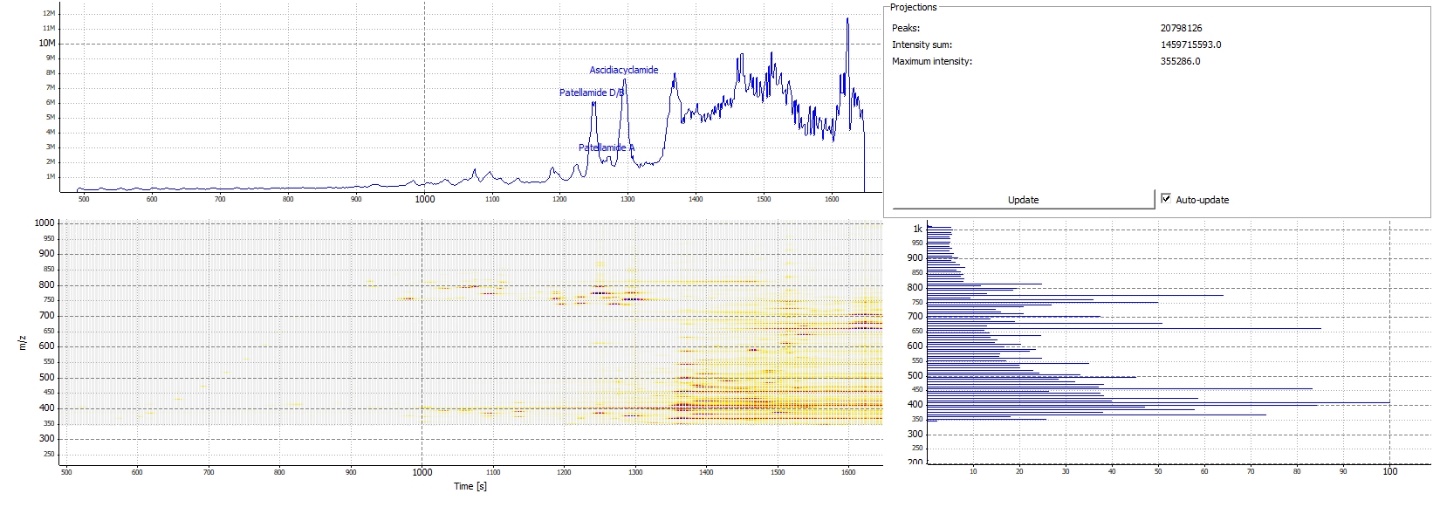

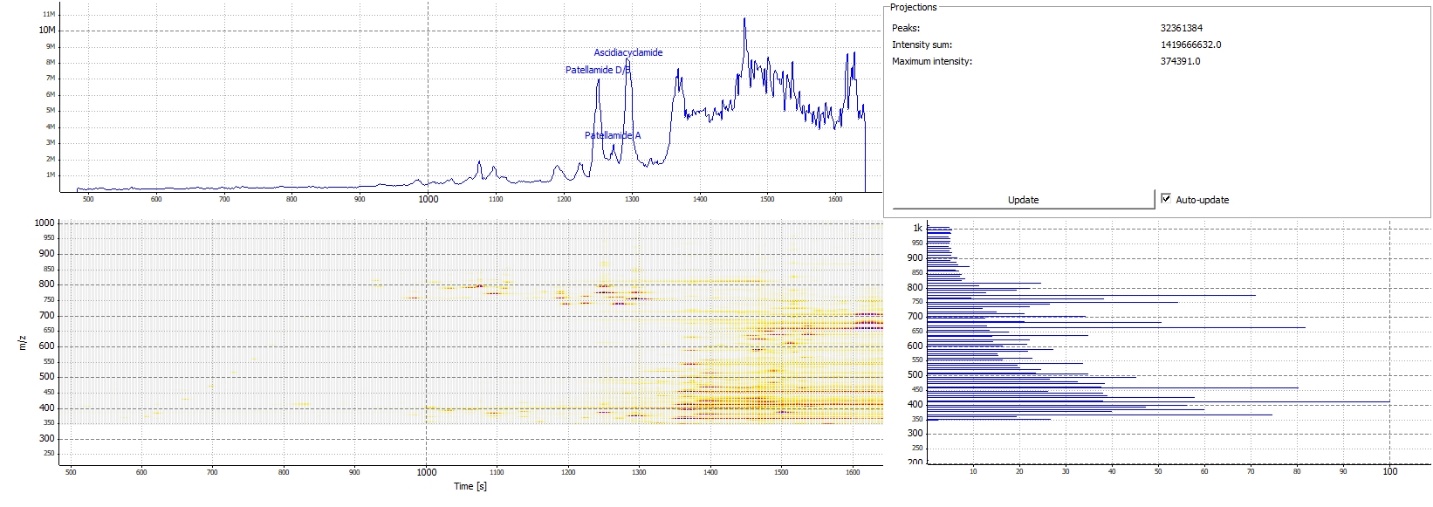


**Figure S26.** HPLC-MS data of two samples of the water extracted from the cloacal cavity without addition of copper, measured at the Sciex TripleTOF 5600 system.


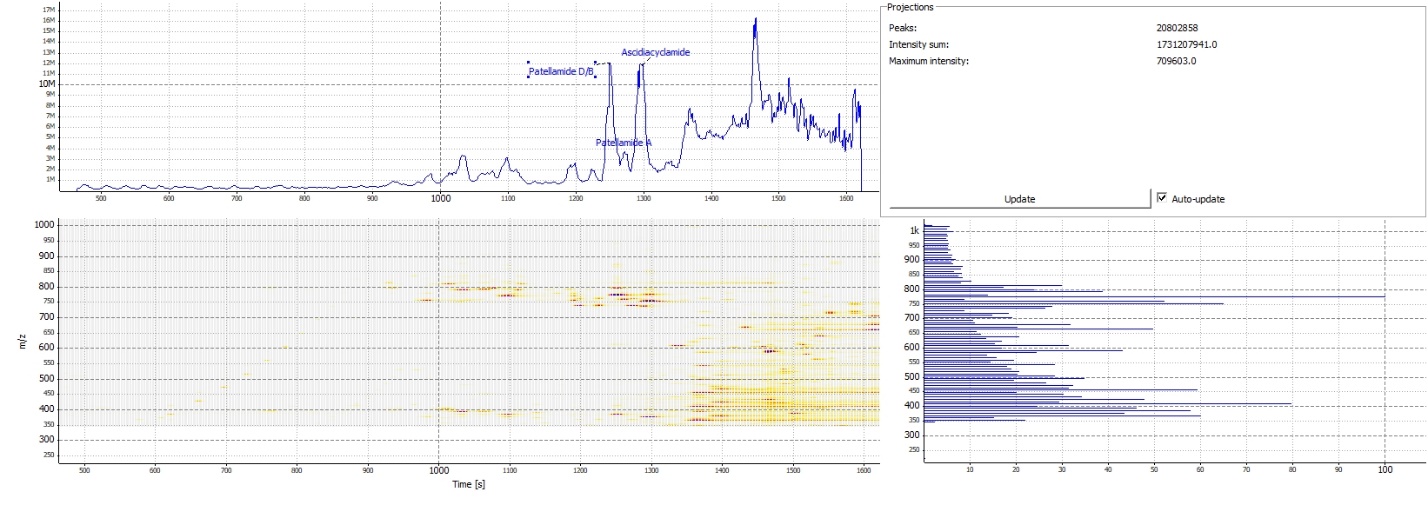


**Figure S27.** HPLC-MS data of the water extracted from the cloacal cavity with addition of copper, measured at the Sciex TripleTOF 5600 system.


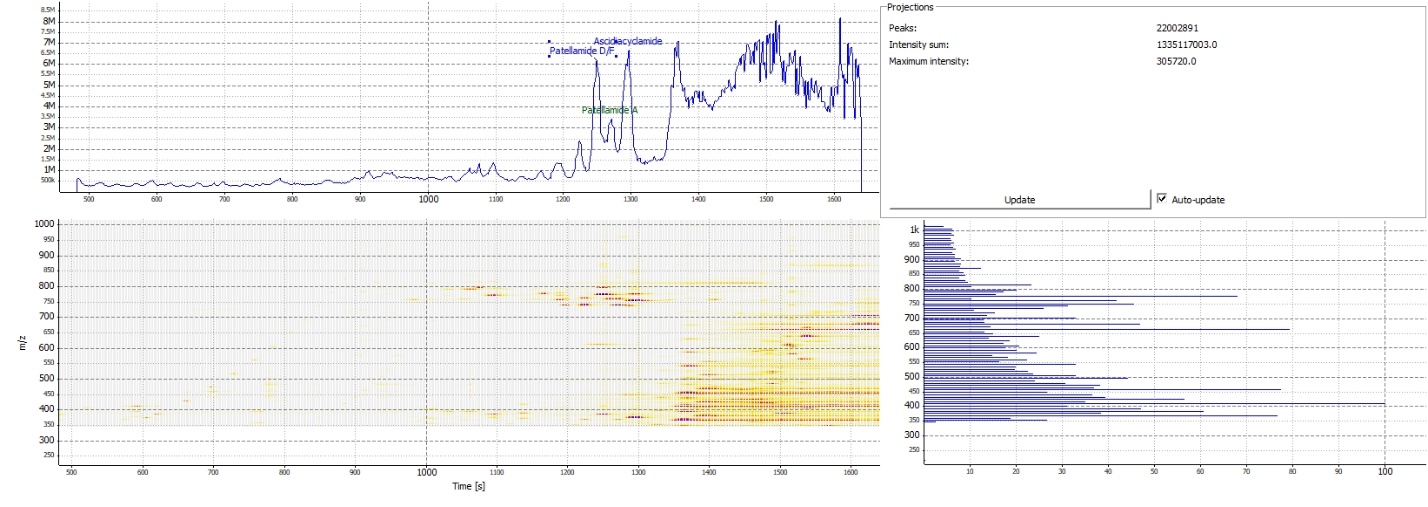


**Figure S28.** HPLC-MS data of the filtered cyanobacteria homogenate extracted from the cloacal cavity without addition of copper, measured at the Sciex TripleTOF 5600 system.


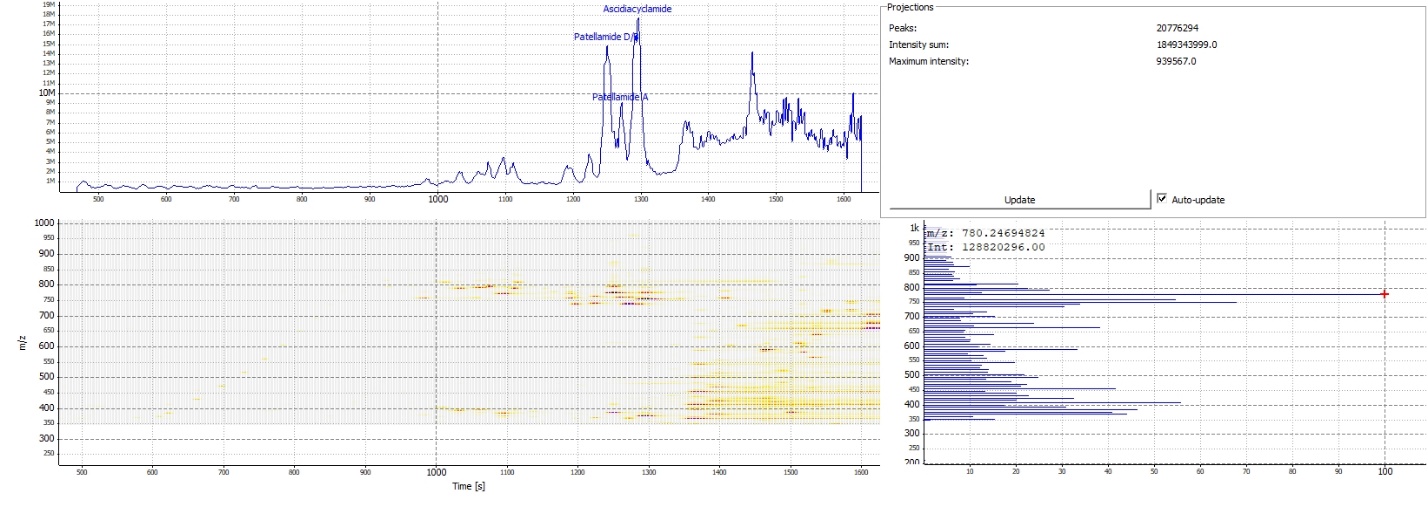

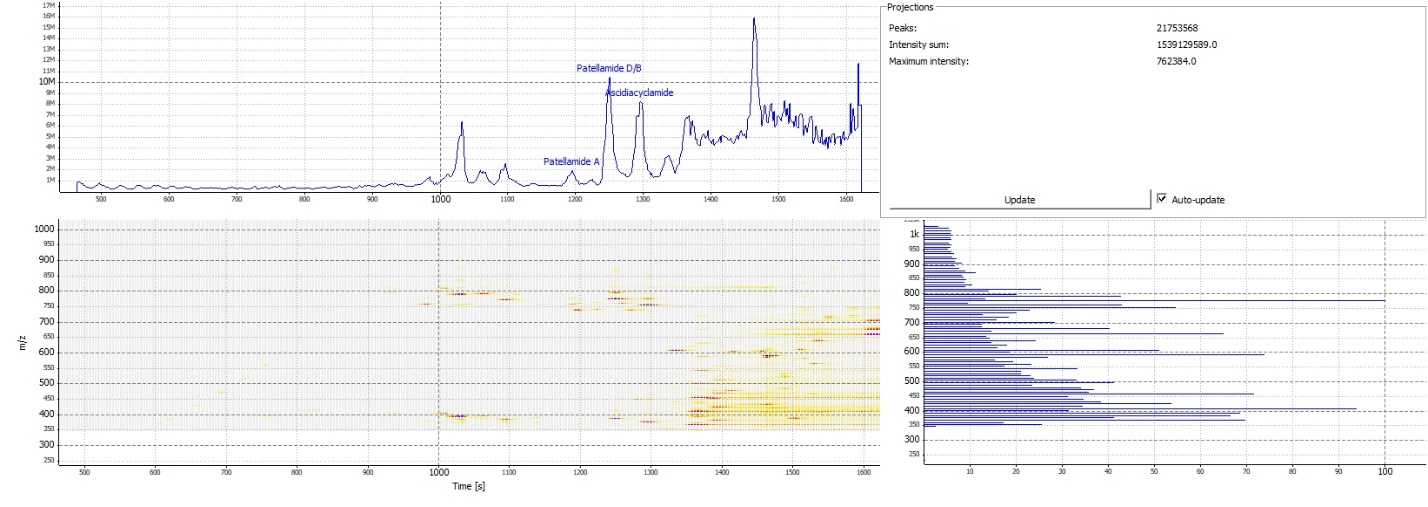


**Figure S29.** HPLC-MS data of two samples of the filtered cyanobacteria homogenate extracted from the cloacal cavity with addition of copper, measured at the Sciex TripleTOF 5600 system.

**Figure S30.** HPLC-MS data of two samples of the water extracted from the cloacal cavity without addition of copper, measured at the Sciex TripleTOF 6600 system.


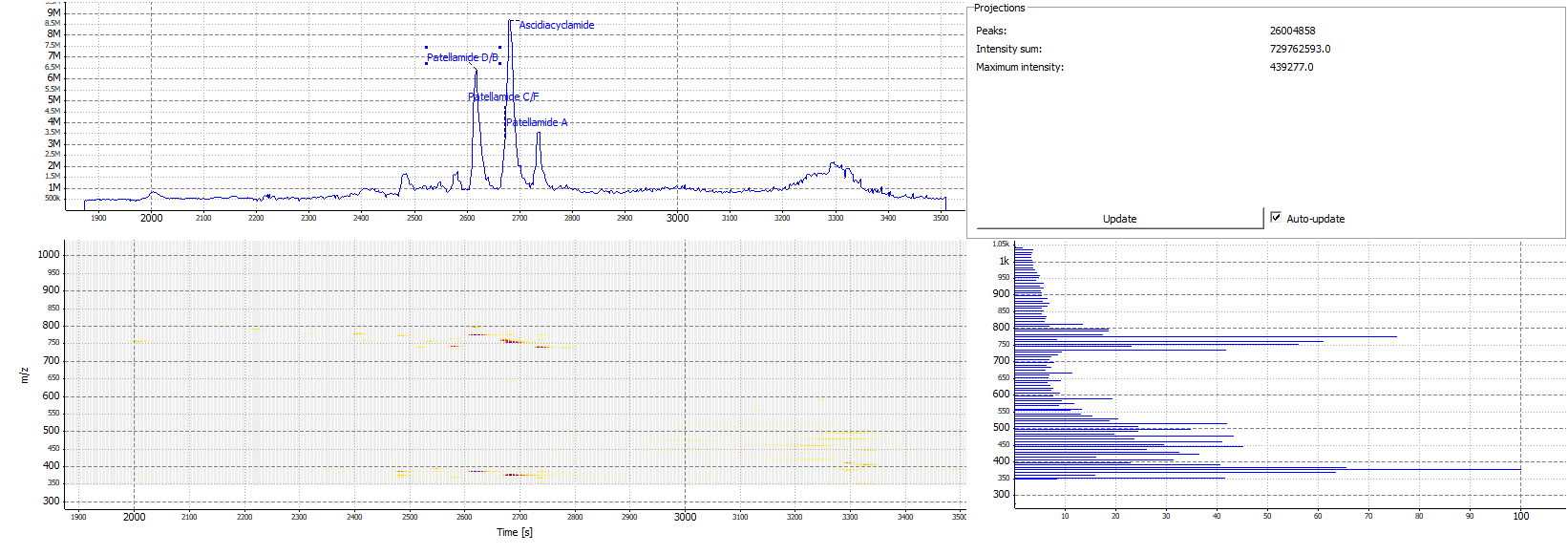

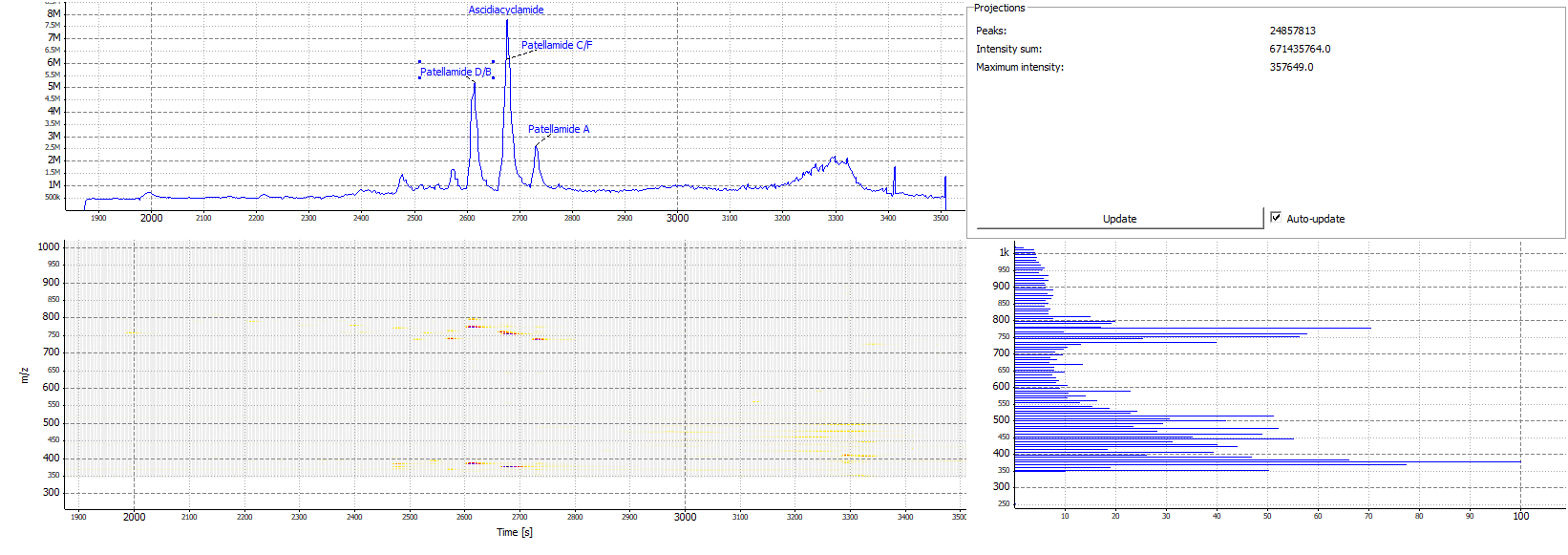

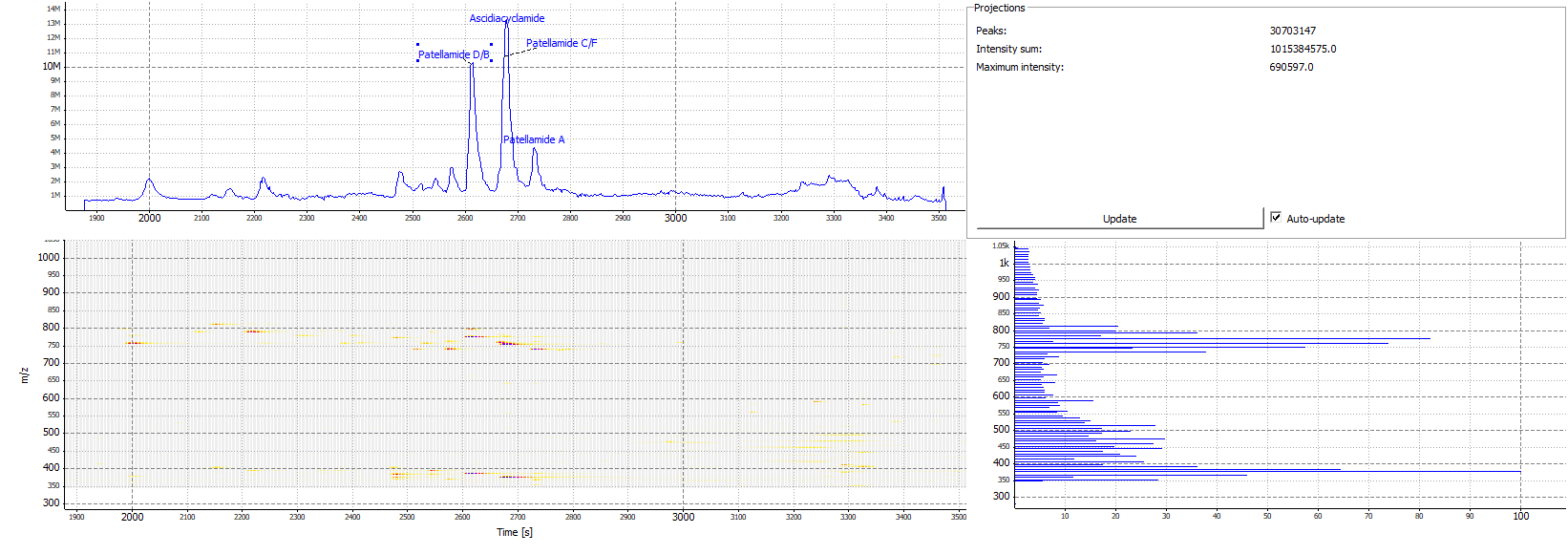

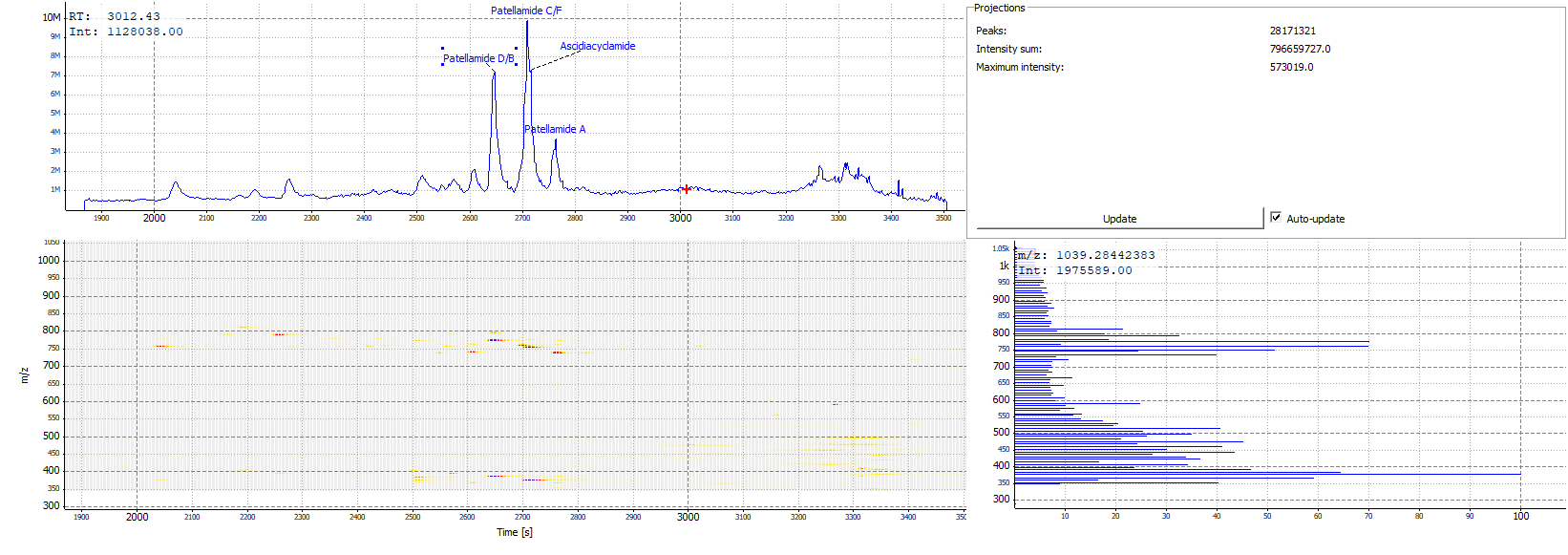


**Figure S31**. HPLC-MS data of two samples of the water extracted from the cloacal cavity with addition of copper, measured at the Sciex TripleTOF 6600 system.


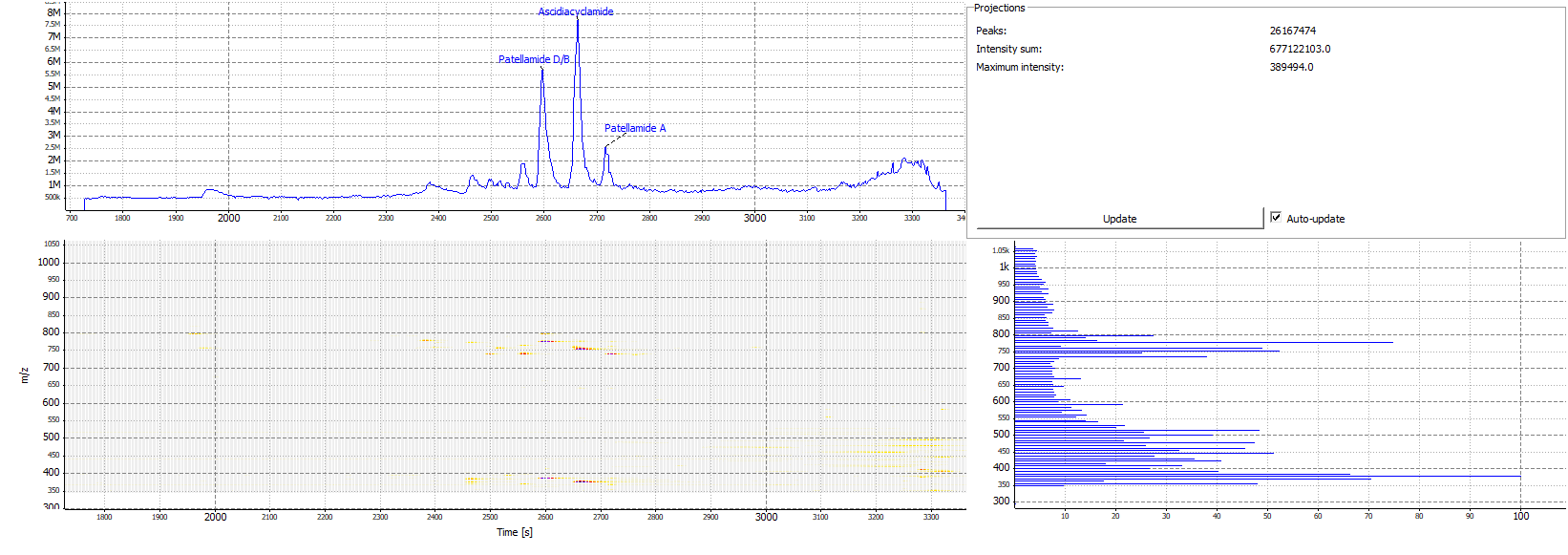


**Figure S32**. HPLC-MS data of the filtered cyanobacteria homogenate extracted from the cloacal cavity without addition of copper, measured at the Sciex TripleTOF 6600 system.


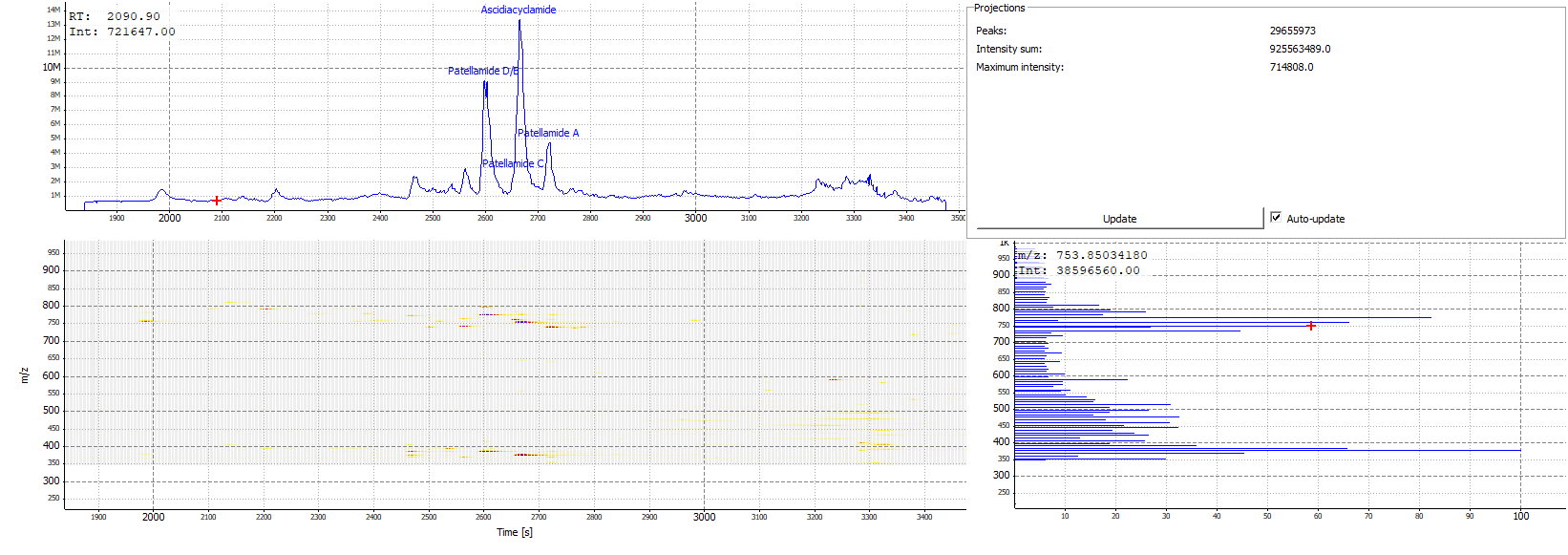

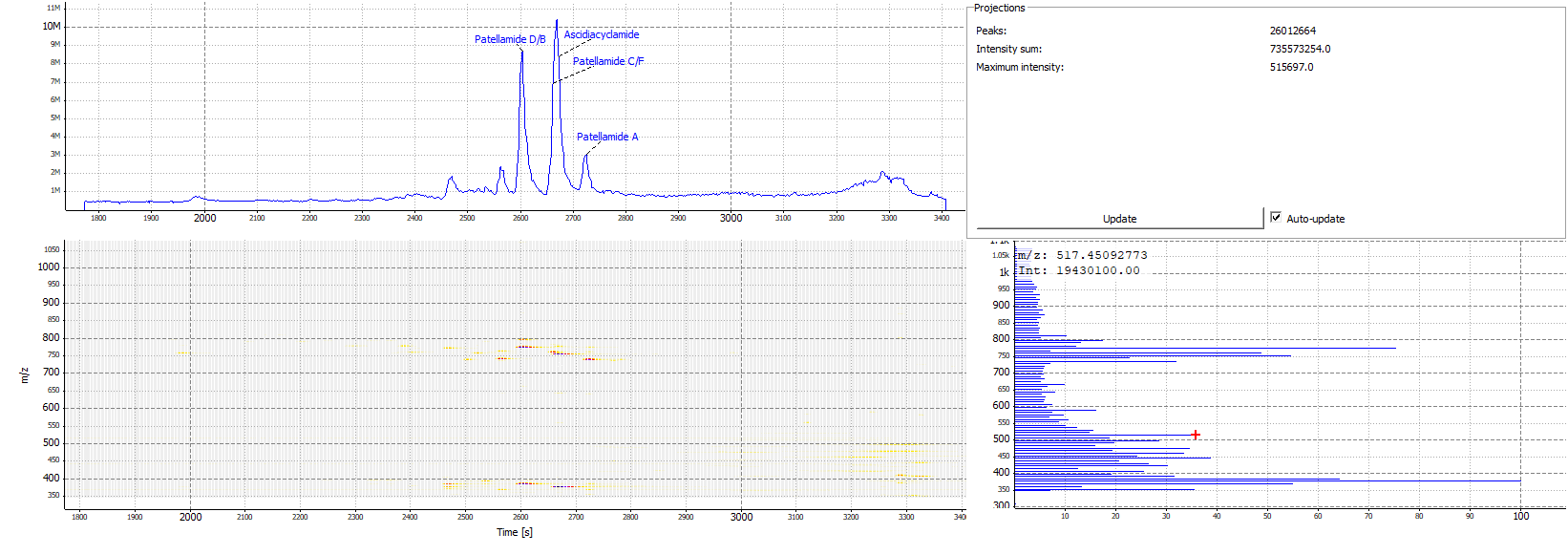


**Figure S33.** HPLC-MS data of two samples of the filtered cyanobacteria homogenate extracted from the cloacal cavity with addition of copper, measured at the Sciex TripleTOF 6600 system.

## **Patellamide membrane permeability (PAMPA, Caco)**

**Table S5:** Raw data of the PAMPA and Caco-2 measurements.

|  |  | |  | |  | |  |  | | | quiniine | | 1.05E-05 (duplicate) | | |  | | **6/25/24** | | quiniine | | 1.82E-5 (triplicate)  **PAMPA without copper** | | |  | | **7/17/24** | | **PAMPA with copper** | |  | |  | |
| --- | --- | --- | --- | --- | --- | --- | --- | --- | --- | --- | --- | --- | --- | --- | --- | --- | --- | --- | --- | --- | --- | --- | --- | --- | --- | --- | --- | --- | --- | --- | --- | --- | --- | --- |
|  |  | |  | |  | |  | **Caco-2 cell** | | | atenolol | | 1.58E-06 (duplicate) | | |  | | **PAMPA** | | atenolol | | 2.75E-6 (triplicate) | | |  | | **PAMPA** | | quinidine | | 1.30E-5 (single) | | | |
| Substance | MW | selected single ion (m/z) | | LC/MS retention time (min)* | | Papp (cm/s) | | | recovery (%) | Papp (cm/s) | | recovery (%) | | Caco-2 note | Papp (cm/s) | | recovery (%) | | Papp (cm/s) | | recovery (%) | | Papp (cm/s) | recovery (%) | | Papp (cm/s) | | recovery (%) | | Papp (cm/s) | | recovery (%) | |  |
| Asc | 757.0 | 757.4 | | 7.84 | | 7.75E-06 | | | 97 | 8.70E-06 | | 104 | | Caco2 passage #54 | 2.53E-05 | | 30 | | 2.09E-05 | | 35 | | 1.77E-05 | 36 | | 9.51E-06 | | 62 | | 9.12E-06 | | 61 | |  |
| PatC | 777.0 | 777.3 | | 7.42 | | 1.28E-05 | | | 111 | 1.35E-05 | | 111 | | Caco2 passage #54 | 1.06E-04 | | 46 | | 2.82E-05 | | 32 | | 2.21E-05 | 37 | | 2.69E-05 | | 45 | | 2.00E-05 | | 42 | |  |
| PatA | 742.0 | 777.3 | | 7.42 | | 5.38E-06 | | | 89 | 5.62E-06 | | 105 | | Caco2 passage #54 | 3.27E-05 | | 41 | | 2.69E-05 | | 41 | | 2.92E-05 | 43 | | 1.99E-05 | | 47 | | 2.71E-05 | | 51 | |  |

## **Patellamide bioactivity towards *Nannochloropsis spp.***

**Figure S34.** Change of the dark-adapted maximum chlorophyll fluorescence of *Nannochloropsis gaditana* after exposing it to different compounds, first series of measurements.

**Figure S35.** Change of the dark-adapted maximum chlorophyll fluorescence of *Nannochloropsis gaditana* after exposing it to different compounds – values relative to the parameter of the blank, second series of measurements.

**Figure S36.** Change of the dark-adapted maximum chlorophyll fluorescence of *Nannochloropsis salina* after exposing it to different compounds – absolute values given. The samples Copper 0/+ correspond to 1/2 ppm of copper in solution. AscCu1 and AscCu

**References**

[1] M. J. Frisch, G. W. Trucks, H. B. Schlegel, G. E. Scuseria, M. A. Robb, J. R. Cheeseman, G. Scalmani, V. Barone, G. A. Petersson, H. Nakatsuji, X. Li, M. Caricato, A. V. Marenich, J. Bloino, B. G. Janesko, R. Gomperts, B. Mennucci, H. P. Hratchian, J. V. Ortiz, A. F. Izmaylov, J. L. Sonnenberg, Williams, F. Ding, F. Lipparini, F. Egidi, J. Goings, B. Peng, A. Petrone, T. Henderson, D. Ranasinghe, V. G. Zakrzewski, J. Gao, N. Rega, G. Zheng, W. Liang, M. Hada, M. Ehara, K. Toyota, R. Fukuda, J. Hasegawa, M. Ishida, T. Nakajima, Y. Honda, O. Kitao, H. Nakai, T. Vreven, K. Throssell, J. A. Montgomery Jr., J. E. Peralta, F. Ogliaro, M. J. Bearpark, J. J. Heyd, E. N. Brothers, K. N. Kudin, V. N. Staroverov, T. A. Keith, R. Kobayashi, J. Normand, K. Raghavachari, A. P. Rendell, J. C. Burant, S. S. Iyengar, J. Tomasi, M. Cossi, J. M. Millam, M. Klene, C. Adamo, R. Cammi, J. W. Ochterski, R. L. Martin, K. Morokuma, O. Farkas, J. B. Foresman, D. J. Fox, Wallingford, CT, **2016**.

[2] aP. J. Hay, W. R. Wadt, *J. Chem. Phys.* **1985**, *82*, 299-310; bW. R. Wadt, P. J. Hay, *J. Chem. Phys.* **1985**, *82*, 284-298; cJ. Zheng, X. Xu, D. G. Truhlar, *Theor. Chem. Acc.* **2010**, *128*, 295-305; dI. Shimizu, Y. Morimoto, G. Velmurugan, T. Gupta, S. Paria, T. Ohta, H. Sugimoto, T. Ogura, P. Comba, S. Itoh, *Chem. Eur. J.* **2019**, *25*, 11157-11165.

[3] B. Ravel, M. Newville, *J. Synchrotron Rad.* **2005**, *12*, 537-541.

[4] A. Tenderholt, B. Hedman, K. O. Hodgson, in *AIP Conference Proceedings*, **2007**, pp. 105-107.

[5] G. N. George, I. J. Pickering, Stanford Synchrotron Radiation Laboratory, Stanford, CA, USA, **1995**.

[6] F. D. V. J. J. Kas, J. J. Rehr, C. D. Pemmaraju, T. S. Tan, *J. Synchrotron Rad.* **2021**.

[7] (Ed.: L. Invitrogen), <https://tools.thermofisher.com/content/sfs/manuals/mp01150.pdf>, **2006**.

[8] N. M. Mangan, A. Flamholz, R. D. Hood, R. Milo, D. F. Savage, *Proc. Natl. Acad. Sci. USA* **2016**, *113*, E5354-5362.

[9] M. Kühl, L. Behrendt, E. Trampe, K. Qvortrup, U. Schreiber, S. M. Borisov, I. Klimant, A. W. Larkum, *Front. Microbiol.* **2012**, *3*, 402.

[10] R. S. J. Allen B. Williams, *Cancer Lett.* **1993**, 97-102.

[11] aK. S. Kumar, Y.-S. Han, K.-S. Choo, J.-A. Kong, T. Han, *J. Toxicol.* **2009**, *1*, 17-23; bH. A. Baumann, L. Morrison, D. B. Stengel, *Ecotoxicol Environ Saf* **2009**, *72*, 1063-1075; cA. J. Miao, W. X. Wang, P. Juneau, *Environ. Toxicol. Chem.* **2005**, *24*, 2603-2611.

[12] L. A. Morris, M. Jaspars, in *Biodivers.*, **2000**, pp. 140-166.

[13] E. Sevin, L. Dehouck, A. Fabulas-da Costa, R. Cecchelli, M. P. Dehouck, S. Lundquist, M. Culot, *J. Pharmacol. Toxicol. Methods* **2013**, *68*, 334-339.

1. The text and Figures in this supporting information have in parts been taken or adapted from the PhD thesis of Philipp Baur (Universität Heidelberg, 2023) [↑](#footnote-ref-1)
